# Single-cell resolved imaging reveals intra-tumor heterogeneity in glycolysis, transitions between metabolic states, and their regulatory mechanisms

**DOI:** 10.1016/j.celrep.2021.108750

**Published:** 2021-02-16

**Authors:** Hiroshi Kondo, Colin D.H. Ratcliffe, Steven Hooper, James Ellis, James I. MacRae, Marc Hennequart, Christopher W. Dunsby, Kurt I. Anderson, Erik Sahai

**Affiliations:** 1 Tumor Cell Biology Laboratory, The Francis Crick Institute, London, NW1 1AT, UK; 2 Metabolomics Science Technology Platform, The Francis Crick Institute, London, NW1 1AT, UK; 3 p53 and Metabolism Laboratory, The Francis Crick Institute, London, NW1 1AT, UK; 4 Photonics Group, Physics Department, Imperial College London, London, SW7 2AZ, UK; 5 Crick Advanced Light Microscopy Facility, The Francis Crick Institute, London, NW1 1AT, UK

**Keywords:** FRET imaging, intra-tumor heterogeneity, tumor metabolism, intravital imaging, cofilin, PI3K signaling, breast cancer

## Abstract

Inter-cellular heterogeneity in metabolic state has been proposed to influence many cancer phenotypes, including responses to targeted therapy. Here, we track the transitions and heritability of metabolic states in single PIK3CA mutant breast cancer cells, identify non-genetic glycolytic heterogeneity, and build on observations derived from methods reliant on bulk analyses. Using fluorescent biosensors *in vitro* and in tumors, we have identified distinct subpopulations of cells whose glycolytic and mitochondrial metabolism are regulated by combinations of phosphatidylinositol 3-kinase (PI3K) signaling, bromodomain activity, and cell crowding effects. The actin severing protein cofilin, as well as PI3K, regulates rapid changes in glucose metabolism, whereas treatment with the bromodomain inhibitor slowly abrogates a subpopulation of cells whose glycolytic activity is PI3K independent. We show how bromodomain function and PI3K signaling, along with actin remodeling, independently modulate glycolysis and how targeting these pathways affects distinct subpopulations of cancer cells.

## Highlights

• Glucose biosensor reveals inter-cellular metabolic heterogeneity *in vivo*
• Single-cell dose response curves reveal variable coupling of PI3K to glycolysis
• Regulation of cofilin couples increasing cell density to metabolic changes
• Metabolic state is heritable through mitosis

## Graphical Abstract

**Figure:**
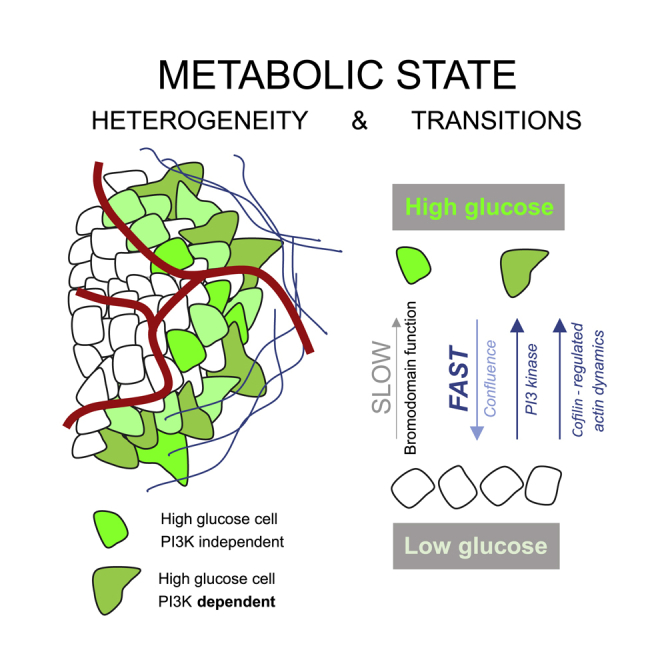

## Teaser

Kondo et al. reveal metabolic heterogeneity in breast cancer by using biosensors, including variable coupling of PI3K to glycolysis. Glucose levels are linked to cell density by cofilin, enabling increased uptake by migrating cells. High-glucose cells grow slowly without pyruvate but are rescued by other cancer cells or stromal fibroblasts.

## Introduction

Intra-tumor heterogeneity presents a challenge for our understanding of cancer biology and cancer therapy. Intra-tumor heterogeneity can manifest at many levels, including mutational and structural changes in the genome, epigenetic variation in chromatin modifications, and local environmental contexts (Dagogo-Jack and Shaw, 2018). Different cancer cells within a tumor can exist in different metabolic states (Lawson et al., 2018). However, metabolic intra-tumor heterogeneity and cellular transitions between metabolic states are difficult to observe. Imaging modalities such as ^18^F-FDG PET/CT and hyper-polarized magnetic resonance imaging (MRI) can distinguish between metabolic states in tumors, but they lack the resolution to study heterogeneity at the cellular level (André et al., 2019; Bertero et al., 2019; Di Leo et al., 2018; Janku et al., 2018). Heterogeneity at this level and switching between metabolic states present a challenge to targeted therapies, including those targeting metabolic regulation directly and those targeting upstream signaling that controls metabolic programs (Hulea et al., 2018).

Glycolysis in tumors is frequently upregulated, leading to increased biosynthesis of metabolic intermediates required for cell proliferation, and may be driven by increased phosphatidylinositol 3-kinase (PI3K) signaling (Hoxhaj and Manning, 2020). In normal physiology, PI3K signaling couples environmental cues to changes in cellular metabolism and acts by AKT to enhance glycolytic flux (Fruman et al., 2017). However, in tumors, PI3K signaling is elevated either through the acquisition of activating mutations, inactivating mutations in the negative regulatory phosphatase PTEN, or as a result of increased receptor tyrosine kinase signaling (Ciruelos Gil, 2014; Razavi et al., 2020). The high prevalence of PI3K activation in tumors has led to the development of PI3K inhibitors, with recent regulatory approval for two PI3K inhibitors in estrogen receptor positive (ER+ve) breast cancer (André et al., 2019; Di Leo et al., 2018; Janku et al., 2018). The efficacy of PI3K inhibitors is thought to be driven, at least in part, by inhibiting PI3K-dependent steps of the glycolytic pathway and limiting glucose flux (Juric et al., 2018, 2019), with reduced fluorodeoxyglucose-positron emission tomography (FDG-PET) uptake observed in ER+ve breast cancer patients treated with the PI3K inhibitor alpelisib (Juric et al., 2018). However, PI3K inhibitors rarely lead to complete clinical responses, with partial responses and stable disease being more typical outcomes. Both cancer cell extrinsic influences, such as systemic increases in insulin, and inter-cellular heterogeneity between cancer cells are likely to be factors limiting the efficacy of the therapy, with one possibility being that a subset of cells persist under PI3K pathway inhibition by regulating glycolysis through other mechanisms (Hopkins et al., 2018; Liu et al., 2011). They might include the recently reported links between actin dynamics and glycolysis or environmental factors, such as hypoxia and substrate stiffness (Bertero et al., 2019; Chae et al., 2016; Hu et al., 2016; Papalazarou et al., 2020; Park et al., 2020).

The development of fluorescent biosensors that report on metabolite levels offers an opportunity to study heterogeneous and fluctuating metabolic states with single-cell resolution (Chennell et al., 2016; Gross et al., 2019; Hung et al., 2017; Imamura et al., 2009; Takanaga et al., 2008; Zhao et al., 2015). In this work, we pursue an integrated approach by using fluorescence biosensors, metabolomics, and intravital imaging to describe heterogeneity in metabolic states in ER+ve breast cancer with single-cell resolution.

## Results

### Breast cancer cells exhibit heterogeneity in metabolic state even under controlled culture conditions

**Figure 1:**
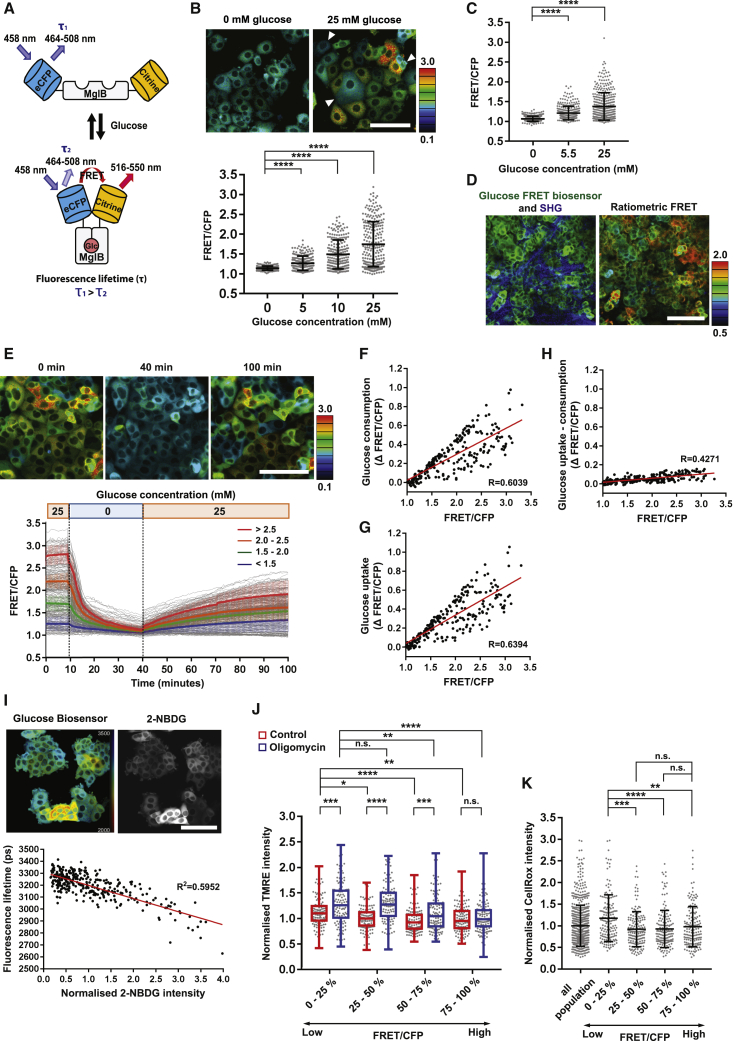
Breast cancer cells exhibit heterogeneity in metabolic state even under controlled culture conditions (A) Schematic representation of the conformational shift of the glucose FRET biosensor upon binding of intracellular glucose. (B) Intracellular glucose levels of MCF-7 cells cultured in 0, 5, 10, or 25 mM glucose media for 1 h. White arrows indicate low-glucose-concentration cells in high-glucose media. Data are shown as mean ± SD; n > 200 cells from 3 independent experiments. Scale, 100 μm. (C) Intracellular glucose levels of MCF-7 cells cultured in 0, 5.5, or 25 mM glucose Plasmax media for 1 h under each condition. Data are shown as mean ± SD; n > 200 cells from 3 independent experiments. (D) Intracellular glucose levels of a representative MCF-7 tumor in non-obese diabetic (NOD) severe combined immunodeficiency (SCID) mouse. Fluorescence of glucose FRET biosensor and SHG are shown in green and blue, respectively. Scale, 100 μm. (E) Glucose uptake and consumption rate of MCF-7 cells. Glucose-FRET-biosensor-expressing MCF-7 cells were incubated with 0 mM glucose media at 10 min and re-cultured with 25 mM glucose media at 40 min. Images were taken every 30 s. Data are shown as mean ± SD; n = 234 cells from 3 independent experiments. Scale, 100 μm. (F–H) Glucose consumption, uptake, and uptake-consumption were calculated from the FRET changes of MCF-7 cells. Plots show single-cell tracing of FRET signals and their FRET shifts. n = 234 cells from 3 independent experiments. (I) Multiplexed imaging of FLIM-FRET of glucose FRET biosensor and 2-NBDG intensity in MCF-7 cells. Cells were incubated with 100 μM 2-NBDG for 2 h. The fluorescence lifetime of the glucose FRET biosensor was plotted against the normalized fluorescence intensity of 2-NBDG. n = 313 cells from 3 independent experiments. Scale, 100 μm. (J) TMRE distribution in low- to high-glucose-concentration cells. Cells were incubated with 100 nM TMRE for 10 min, and then the cells were incubated with 2 μM oligomycin for 1 h. Data are shown as boxplots of median with lower to higher quartiles and minimum to maximum whiskers. n > 100 cells from 3 independent experiments. (K) CellRox distribution in low- to high-glucose-concentration cells. Cells were incubated with 5 μM CellRox for 30 min. Data are shown as mean ± SD; n > 150 cells from 3 independent experiments. Statistical significance of glucose FRET biosensor, TMRE, and CellRox were examined by Kolmogorov-Smirnov test. p values are indicated by ns, p > 0.05; ^∗∗^p < 0.01; ^∗∗∗^p < 0.001; and ^∗∗∗∗^p < 0.0001.

To study heterogeneity in metabolic state in breast cancer models, we first used a glucose fluorescence resonance energy transfer (FRET) biosensor based on the bacterial MglB protein (Figure 1A; Takanaga et al., 2008). Binding of glucose to the biosensor changes both the color of emitted light and the fluorescence lifetime (Figure 1A). The biosensor did not affect the growth of cells stably expressing the fusion protein; a glucose-defective sensor was used as a control (Figure S1A). We detected a glucose-dependent FRET signal in MCF-7 breast cancer cells cultured in both DMEM and Plasmax medium that more closely mimics nutrient levels *in vivo* (Figures 1B and 1C; Vande Voorde et al., 2019). Considerable heterogeneity was observed at physiological glucose levels (5 mM glucose in DMEM and 5.5 mM glucose in Plasmax media; Figures 1B and 1C). Furthermore, implantation of these cells into the mammary fat pad of mice revealed inter-cellular heterogeneity in FRET signals (Figure 1D; to aid comparison with the *in vitro* analysis, the image is shown using the same color scale in Figure S1B). To our surprise, we also observed inter-cellular heterogeneity in cells grown under uniform cell culture conditions. Even in media containing high (4.5 g/L) levels of glucose, some cells had very low intracellular glucose level, similar to those observed in glucose-free medium (Figures 1B and S1C). These differences in FRET signal were not related to the expression of biosensor (Figures S1C–S1E). Widespread inter-cellular heterogeneity in glucose concentration was observed even in cells expanded from a single-cell clone, indicating that the source of the heterogeneity is non-genetic (Figure S1F).

The observation of high intracellular glucose suggests faster uptake than utilization but is not sufficient to distinguish between high and low rates of glucose use. To discriminate between these possibilities, we washed cells into glucose-free medium and then, once the biosensor signal had dropped to baseline, returned them to 4.5 g/L glucose medium. Figure 1E and Video S1 show single-cell measurements of the glucose biosensor signal throughout the course of this experiment. We assessed the rate of glucose utilization by measuring the initial slope of the drop in glucose-dependent FRET signal following wash into glucose-free media (Figure 1F). The cells with the fastest rate of glucose use had high glucose levels prior to the medium switch. Exponential fitting and derivation of the t_1/2_ for glucose levels to drop also confirmed the inference that high FRET cells used glucose faster (Figure S1G). Treatment with koningic acid to inhibit GAPDH confirmed that the drop in glucose biosensor signal was predominantly due to glycolysis (Figures S1H and S1I). Moreover, subsequent glucose wash-in in the presence of koningic acid boosted the glucose FRET signal to higher levels than those of untreated cells. The rate of glucose uptake from the medium could be inferred by adding the rate of utilization to the rate of biosensor signal increase following transfer back into high-glucose media (Figures 1E–1G). Cells with a high starting FRET ratio also show high rates of glucose uptake. Similar results were observed in Plasmax medium (Figure S1J). These data argue that cells with a high-glucose biosensor signal have high rates of both glucose uptake and consumption. Their high signal at steady state is explained by a faster rate of glucose uptake and not a slower rate of glucose use. We confirmed this finding by combining biosensor imaging with uptake of fluorescent 2-NBDG. This required further biosensor engineering due to the overlapping fluorescence of the 2-NBDG with the acceptor fluorophore in the FRET biosensor construct. FRET can be measured by a reduction in the fluorescence lifetime of the donor fluorophore. To avoid spectral overlap of citrine and 2-NBDG, we swapped the donor fluorophore from eCFP to mTurquoise2 and the acceptor from citrine to sReACh (Martin et al., 2018), which can accept energy from mTurquoise2 but is not fluorescent itself. As expected, high glucose in the extracellular medium reduced the fluorescence lifetime of mTurquoise2 (Figures S1K and S1L). Furthermore, we found that shorter single-cell fluorescence lifetimes correlate with high 2-NBDG uptake (Figure 1I), confirming that a high-glucose biosensor signal reflects fast glucose uptake.

Video S1. Single-cell measurements of the glucose biosensor signal throughout glucose wash-out/wash-in analysis in sub-confluent condition, related to Figures 1E–1H Glucose FRET biosensor-expressing MCF-7 cells were incubated with 25 mM glucose media from 0 to 10 minutes. The media was replaced with 0 mM glucose media at 10 minutes and the cells were incubated for 30 minutes. The cells were re-cultured in 25 mM glucose media from 40 minutes. Images were taken every 30 s. Scale = 100 μm.

To obtain more information on the metabolic state of cells with high and low intracellular glucose concentrations, we combined imaging of the glucose biosensor with other metabolic probes. TMRE fluorescence, which reports on mitochondrial membrane potential, a requirement for ATP production through OXPHOS, was lower in cells with high intracellular glucose levels (Figure 1J, upper quartile [75%–100%]). Treatment with oligomycin, an inhibitor of ATP synthase that prevents the utilization of protons in the mitochondrial inner membrane space, boosted the TMRE signals in all cells except the high-glucose FRET cells (Figure 1J). These data argue the cells with high intracellular glucose levels have lower proton transport and mitochondrial membrane potential. Combining glucose biosensor imaging with CellRox to detect reactive oxygen species (ROS) revealed that low-FRET cells (bottom 25%) had higher ROS levels, which is also consistent with increased mitochondrial function (Figure 1K). Together, these data show that the glucose FRET biosensor reveals inter-cellular heterogeneity in the relative utilization of glycolysis and OXPHOS even within a cancer cell line maintained under uniform cultured conditions.

### Heritability and transitions between high- and low-glucose states

**Figure 2:**
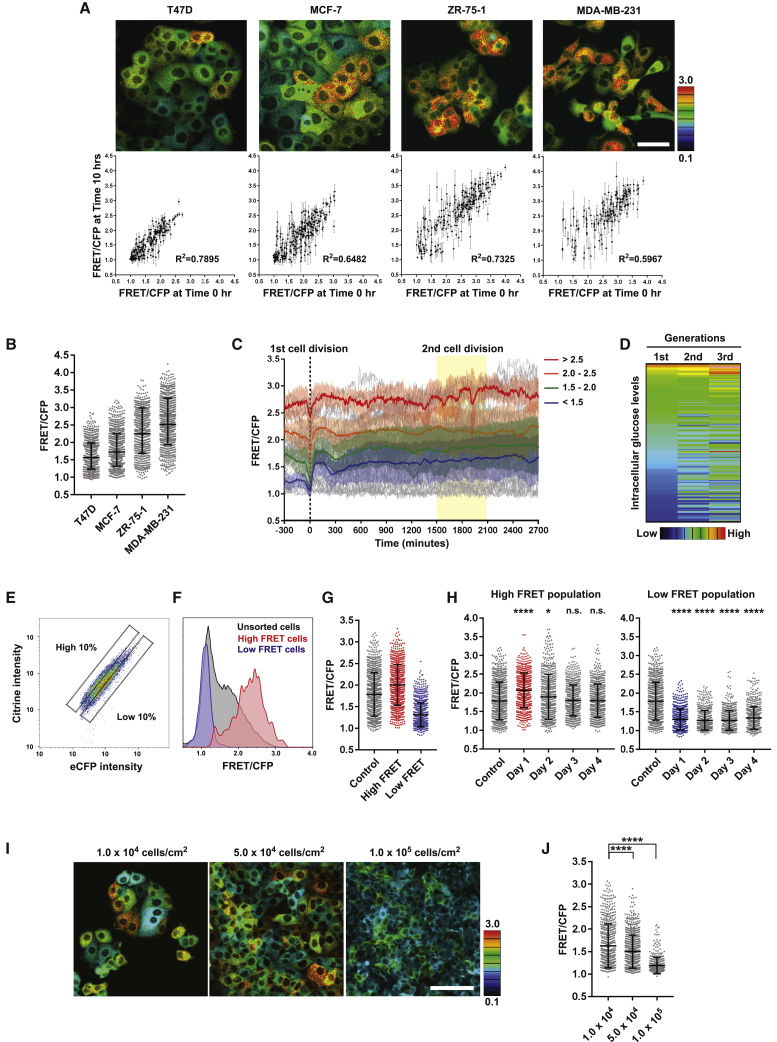
Heritability and transitions between high and low glucose states (A) Stability of heterogeneous intracellular glucose levels in breast cancer cell lines. Glucose-FRET-biosensor-expressing breast cancer cell lines were imaged for 10 h. Following cell segmentation, glucose levels were tracked, and glucose FRET signals at time (T) = 0 h against signal at T = 10 h were plotted with the standard deviation over 10 h. n > 150 cells from 3 independent experiments. Scale, 50 μm. (B) FRET distribution of intracellular glucose levels in four breast cancer cell lines. n > 350 cells from at least 3 independent experiments. (C) Heritability of heterogeneous intracellular glucose levels in MCF-7 cells. Glucose-FRET-biosensor-expressing MCF-7 cells were imaged for 3,000 min with 5 min-intervals. Following cell segmentation, intracellular glucose levels were tracked in individual cells and relabeled to the first mitosis at T = 0 min. n = 100 cells from 3 independent experiments. (D) Heatmap of intracellular glucose levels of MCF-7 cells at first, second, and third generations. (E) Cell sorting results of high- and low-glucose-concentration MCF-7 cells. The panel shows the FRET distribution of glucose biosensor-expressing MCF-7 cells. Cells were isolated from the dashed squares. (F) FRET distribution of FACS-isolated high- and low-glucose-concentration cells. (G) Imaging intracellular glucose levels of FACS-isolated high- and low-glucose-concentration cells using confocal microscopy. n > 500 cells from at least 3 independent experiments. (H) Stability and heritability of FACS-isolated high- and low-glucose-concentration cells. High- and low-glucose cells were isolated by FACS, and intracellular glucose levels were traced for 4 continuous days. n > 500 cells from at least 3 independent experiments. (I and J) Intracellular glucose levels with different cell densities. The cells were cultured in three different cell densities for 24 h, and then intracellular glucose levels were measured by ratiometric FRET imaging. n > 400 cells from at least 3independent experiments. Scale, 100 μm. Data are shown as mean ± SD. Statistical significance was examined by Kolmogorov-Smirnov test. p values are indicated by ns, p > 0.05; ^∗^p < 0.05; ^∗∗∗∗^p < 0.0001.

We next sought to determine inter-cellular heterogeneity in glycolytic state in other breast cancer cell lines and whether it fluctuated over time. Three other breast cancer cell lines spanning both ER+ve and triple-negative sub-types were engineered to express the glucose FRET biosensor and were imaged for 10 h (Video S2). As with MCF-7 cells, inter-cellular heterogeneity in glucose concentration was observed in T47D, ZR-75-1 and MDA-MB-231 cells in 4.5 g/L glucose (Figures 2A, 2B, and S2A). The variance of the biosensor signal over the 10-h period was calculated for each cell (Figure S2B). This metric gives an indication of short-term metabolic plasticity. Figure 2A shows that the aggressive triple-negative MDA-MB-231 cell line had the highest variance. The variance in single MCF-7 and T47D cells was small relative to the mean value for that cell and was also small relative to the range of means, indicating that although intracellular glucose concentrations fluctuate, cells do not switch between high and low-glycolysis states within a 10-h period. This finding was confirmed by plotting the glucose concentration at t = 0 and t = 10 h (Figure 2A). In all cell lines, there was a strong positive correlation, although, once again, MDA-MB-231 cells had the largest changes in glucose-dependent FRET values over the time period. To determine if glycolytic state was heritable through mitosis, we undertook 50-h time-lapse imaging, which enabled two mitoses to be captured for most cells. Glucose concentration traces for every cell were “synchronized” *in silico* to the time of the first mitosis, with one track giving rise to two tracks corresponding to the daughter cells after mitosis (Figure 2C). For ease of visualization, cells were sub-divided depending on their glucose concentration at the first mitosis, and the mean and variance of each category was color coded—which included all progeny resulting from the initial mitosis. The resulting traces were broadly flat, indicating that intracellular glucose concentration remains consistent over 40 h and one or two mitoses. Plotting the glucose FRET signal of the parent cell against its daughter cells and its granddaughter cells revealed that the intracellular glucose concentration of daughter cells was closely correlated with that of the parent cell (Figures 2D and S2C). The strength of this correlation declined if the intracellular glucose concentration of granddaughter and grandparent cells were compared. These data demonstrate that inter-cellular heterogeneity in glucose utilization is a heritable trait, at least over one or two cell cycles. Although metabolic states appear stable over the course of a couple of cell divisions, single-cell cloning suggested that heterogeneity arose from non-genetic means (Figure S1F).

Video S2. Imaging glucose biosensor signals in four breast cancer cell lines, related to Figures 2A and 2B Time-lapse imaging of intracellular glucose level of breast cancer cell lines. Glucose FRET biosensor-expressing T47D, MCF-7, ZR-75-1, and MDA-MB-231 cells were imaged for 10 hours every 30 s. Scale = 100 μm.

We next sought to use fluorescence-activated cell sorting (FACS) to characterize high- and low-glucose cells further. Figures 2E and 2F confirm the feasibility of this approach. We sorted high- and low-glucose populations (Figure 2G) and followed them over several days. In agreement with the time-lapse imaging, these data show that it takes many days for populations of sorted cells to return to the mean (Figure 2H). Furthermore, the transition of high-glucose cells back to the mean is more rapid than the transition of low-glucose cells. Similar results were obtained with T47D and ZR-75-1 cells (Figures S2D–S2G). During the course of these experiments, we noted that the transition from high to low glucose was coincident with an increase in cell density as a result of proliferation. To test if cell density might be responsible for the transition to a low-glucose state, we plated cells at different densities. This test demonstrated that high cell density is sufficient to reduce intracellular glucose (Figures 2I and 2J).

### High and low intracellular glucose concentrations indicate relative preference for glycolysis and OXPHOS

**Figure 3:**
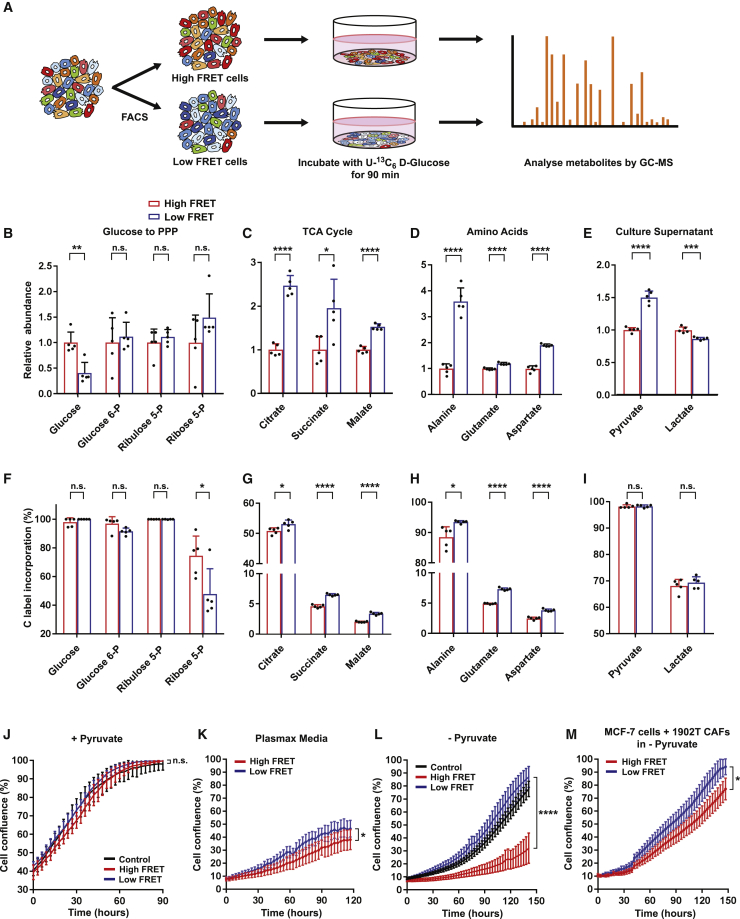
High and low intracellular glucose concentrations indicate relative preference of glycolysis and OXPHOS (A) Schematic representation of metabolomic analysis of FACS-isolated high- and low-glucose cells. High- and low-glucose-concentration cells were isolated from glucose-FRET-biosensor-expressing MCF-7 cells. FACS-isolated high- and low-glucose-concentration cells were incubated with U-^13^C_6_ D-glucose for 90 min, and then extracted metabolites were analyzed using GC-MS. (B–I) Metabolomic analysis of FACS-isolated high- and low-glucose cells and culture supernatant. Abundance and ^13^C incorporation into metabolites of glycolysis, the PPP, TCA cycle, and amino acids were measured in high and low-glucose cells and associated culture supernatant (n = 5 biological replicates). Abundance is shown relative to high FRET conditions, and label incorporation is shown as percent molecules containing ^13^C atoms. (J–L) FACS-isolated high- and low-glucose MCF-7 cells were cultured in 1 mM pyruvate DMEM (J), Plasmax media (K), or 0 mM pyruvate DMEM (L). Cell confluence was measured every 3 h using IncuCyte S3 Live-Cell Analysis System. n > 10 wells from 3 independent experiments. (M) Growth curve of FACS-isolated high- and low-glucose MCF-7 cells co-cultured with 1902T CAFs in DMEM without pyruvate. n > 10 wells from 3 independent experiments. Data are shown as mean ± SD. Statistical significance was examined by two-tailed unpaired t test. p values are indicated by n.s., p > 0.05; ^∗^p < 0.05; ^∗∗^p < 0.01; ^∗∗∗^p < 0.001; ^∗∗∗∗^;p < 0.0001.

The ability to sort cells with high- and low-glucose-dependent FRET opened up the possibility of performing bulk analyses, in particular metabolomic and biochemical characterization, on populations with distinct metabolic features. Flow cytometry was used to isolate high- and low-glucose-concentration cells that were subsequently cultured in U-^13^C_6_ labeled glucose (Figure 3A). Gas chromatography-mass spectrometry (GC-MS) analysis confirmed that intracellular glucose was efficiently labeled and high FRET signal cells did indeed have more glucose (Figures 3B and 3F). High-glucose cells also showed a higher transit of glucose-derived carbon into the pentose phosphate pathway intermediate ribose 5-phosphate (Figure 3F). In contrast, in low-glucose cells, we observed increased entry of glucose-derived carbon into the tricarboxylic acid (TCA) cycle, as indicated by higher levels of labeled citrate, succinate, and malate (Figures 3C and 3G). We also noted increased labeling of the amino acids alanine, glutamate, and aspartate, supporting a shift toward the TCA cycle for biosynthetic production in low-glucose-concentration cells (Figures 3D and 3H). Increased mitochondrial oxygen consumption rate (OCR) further supports our conclusion that low-glucose cells have higher mitochondrial metabolism and activity (Figure S3A). Together with previous TMRE and 2-NBDG analysis, these data are consistent with a model in which the high-glucose-concentration cells favor aerobic glycolysis and low-glucose FRET cells favor mitochondrial metabolism. Interestingly, both high- and low-glucose-concentration cells grew at equivalent rates in complete media, but high-glucose cells were disadvantaged in the more physiological Plasmax media and in the absence of pyruvate (Figures 3J and 3K). This finding may be linked to either reduced excretion of pyruvate or increased uptake of pyruvate from the media (Figures 3E, 3I, and 3L).

### Inter-cellular heterogeneity in response to PI3K inhibition depends upon bromodomain function

**Figure 4:**
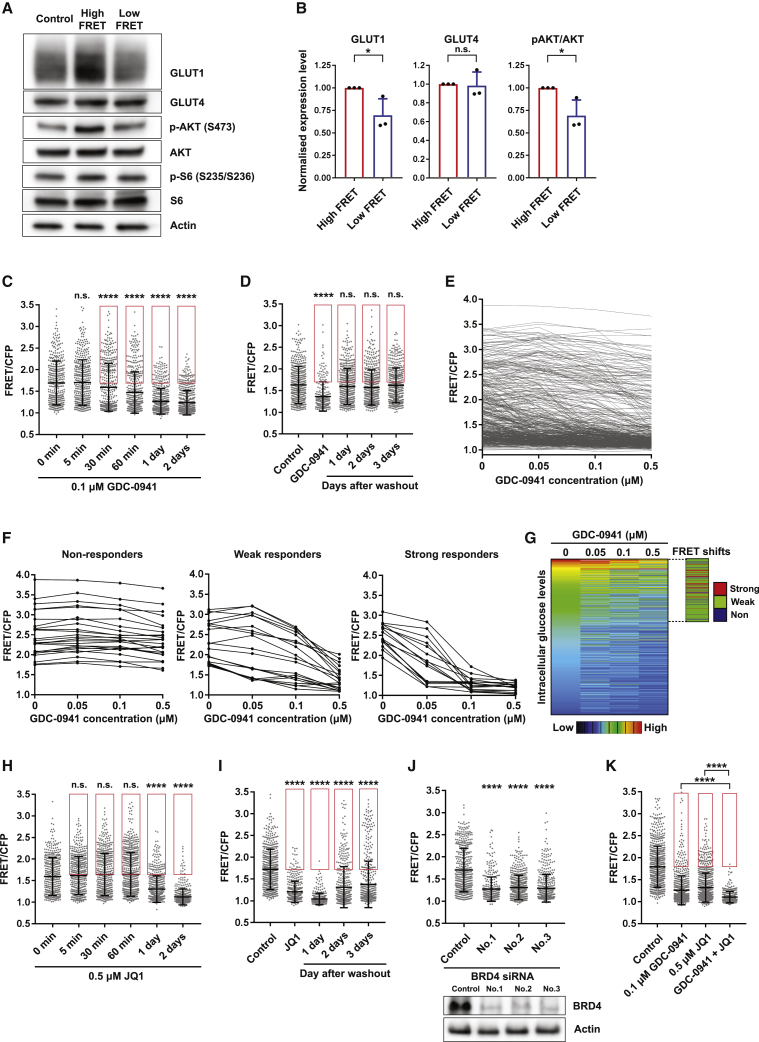
Inter-cellular heterogeneity in response to PI3K inhibition depends upon bromodomain function (A and B) Western blot of metabolic regulators in FACS-isolated high- and low-glucose-concentration cells. Expression and phosphorylation levels of GLUT1, GLUT4, and AKT were quantified from 3 independent blots. (C and D) Time course imaging of intracellular glucose levels after PI3K inhibitor treatment. Intracellular glucose levels were traced after wash out of the inhibitor. The cells indicated by the red square represent inhibitor-resistant high-glucose-concentration cells. n > 200 cells from 3 independent experiments. (E) Time-lapse imaging of intracellular glucose levels of MCF-7 cells treated with increasing PI3K inhibitor (GDC-0941) concentrations. The FRET ratio of cells was measured in 0 μM at 0 h, 0.05 μM at 1 h, 0.1 μM at 2 h, and 0.5 μM at 3 h. n = 450 cells from 3 independent experiments. (F) Intracellular glucose levels of non-, weak, or strong responders to different concentrations of GDC-0941. (G) Heatmap of the FRET ratio of MCF-7 cells in different GDC-0941 concentrations. FRET shifts of non-, weak, or strong responders are presented in blue, green, or red, respectively. (H and I) Time course imaging of intracellular glucose levels after a bromodomain inhibitor (JQ1) treatment. Intracellular glucose levels were traced after wash out of the inhibitor. The cells indicated by the red square represent inhibitor-resistant high-glucose-concentration cells. n > 300 cells from 3 independent experiments. (J) FRET distribution of intracellular glucose levels of MCF-7 cells with BRD4 knockdown. n > 350 cells from 3 independent experiments. (K) The effect of combinatorial inhibition of PI3K and bromodomain on intracellular glucose levels. MCF-7 cells were treated with 0.1 μM GDC-0941 and 0.5 μM JQ1. The cells indicated by the red square represent inhibitor-resistant high-glucose-concentration cells. n > 350 cells from 3 independent experiments. Data are shown as mean ± SD. Statistical significance of western blot (WB) was examined by two-tailed unpaired t test. Statistical significance of glucose FRET biosensor was examined by Kolmogorov-Smirnov test. p values are indicated by ns, p > 0.05; ^∗^p < 0.05; ^∗∗∗∗^p < 0.0001.

Thus far, our data document metabolic heterogeneity with single-cell resolution, but apart from the effect of cell confluence, they do not provide information about the underlying sources of the variation. To gain insights into this information, we exploited our ability to sort high- and low-glucose-concentration cells and combined it with loss-of-function approaches. Western blot analysis revealed that high-glucose-concentration cells had modestly elevated levels of PI3K signaling (as assessed by pS473-AKT) and the glucose transporter GLUT1 but not GLUT4 (Figures 4A and 4B). No significant differences were seen in pS235/S236-S6 levels, consistent with the similar growth rates the two populations displayed in complete media. The association between the high-glucose state and PI3K signaling was not particularly surprising, but nonetheless, we investigated if PI3K signaling was required for the high-glucose FRET signal. Time-lapse imaging revealed that the pan-PI3K inhibitor GDC-0941 reduced glucose-dependent FRET within 30 min (Figure 4C). However, 11% of cells were refractory to the effect of GDC-0941, meaning that their FRET signal remained above the median value of the control population (Figure 4C, drug-refractory cells highlighted with a red box). Simultaneous analysis of an AKT sensor, a fragment of the transcription factor FoxO1 fused to mRuby2 that is cytoplasmic when phosphorylated by AKT (Gross et al., 2019), confirmed that PI3K signaling was effectively inhibited in all cells (Figure S4A). Consistent with previous reports, the effect of GDC-0941 was reversible (Figure 4D), and this refractory population of cells was observed upon treatment with alpelisib or AZD8186, other isoform-specific PI3K inhibitors (Figure S4B; Yang et al., 2016).

It is possible that this refractory population of cells indicates that there is inter-cellular heterogeneity in the coupling of PI3K signaling to glycolysis within MCF-7 cells, with some cells highly sensitive to small changes in the strength of PI3K signaling and others exhibiting only small changes in glycolysis even when PI3K is inhibited. To explore this directly, we performed single-cell resolved dose-response analyses (Figures S4C and S4D). Cells were exposed to increasing concentrations of GDC-0941, and the FRET signal was recorded at each drug concentration (Figure 4E). For illustrative purposes, exemplars of non-responders, weak responders, and strong responders to GDC-0941 are shown in Figure 4F. Figure 4G shows a color-coded summary of the single-cell dose-response data and whether cells were non-, weak, or strong responders. These analyses reveal striking variation in the responsiveness of glucose concentration to PI3K inhibition: in 10% of high-glucose cells the regulation of glycolysis was highly sensitive to small perturbations in PI3K signaling, but 11% of high-glucose cells showed little change in glucose concentration upon complete PI3K inhibition (Figures 4F, 4G, and S4C). Our previous analysis had indicated that cell confluence modulates glucose FRET (Figures 2I and 2J); therefore, we examined the interplay between PI3K inhibition and confluence. Figures S4E–S4H show that high-glucose FRET cells that remain at high confluence are not affected by PI3 kinase inhibition. These data reveal inter-cellular heterogeneity in the coupling of PI3K signaling and glucose concentration and demonstrate that responders and non-responder cells cannot be distinguished on the basis of their initial glucose concentration.

We next sought to identify perturbations that could reduce inter-cellular heterogeneity in the linkage between PI3K signaling and glycolysis. Previous bulk analyses have suggested a role for bromodomains in regulating the expression of upstream regulators of PI3K signaling (Kitajima et al., 2018; Stratikopoulos et al., 2015). Therefore, we combined treatment with PI3K inhibitor and the bromodomain inhibitor JQ1. In contrast to PI3K inhibition, bromodomain inhibition had no effect on glucose-dependent FRET signal over time periods up to 1 h (Figure 4H). After 24 h, JQ1 treatment reduced intracellular glucose levels, although not all cells had glucose concentrations below the median of the control. Furthermore, the effect of JQ1 treatment persisted even after the drug had been removed (Figure 4I). Depletion of BRD4 had a similar effect to JQ1 treatment, suggesting that BRD4 is the most relevant bromodomain target of JQ1 (Figure 4J). Combining bromodomain inhibition with PI3K inhibition abolished the sub-population of cells refractory to high levels of the PI3K inhibitor (Figure 4K). This result was observed at both high and low cell confluence (Figures S4F–S4H). We consistently observed a reduction in the refractory subpopulation for each pairwise combination of PI3K inhibitor (GDC-0941, alpelisib, and AZD5153) and bromodomain inhibitor (JQ1 and AZD5153) tested (Figure S4A). Combinatorial inhibitor treatment also induced high energetic stresses, including low glucose uptake, low mitochondrial membrane potential, and high ROS levels, as well as low ATP production (Figures S4I–S4L). Together, these data uncover considerable heterogeneity in the response of breast cancer cells to PI3K inhibition, even when cultured under uniform conditions. Our data support a model in which cells with high levels of bromodomain function are less sensitive to small changes in PI3K signaling.

### Confluence-dependent control of cofilin activity is a parallel regulator of intracellular glucose concentration

**Figure 5:**
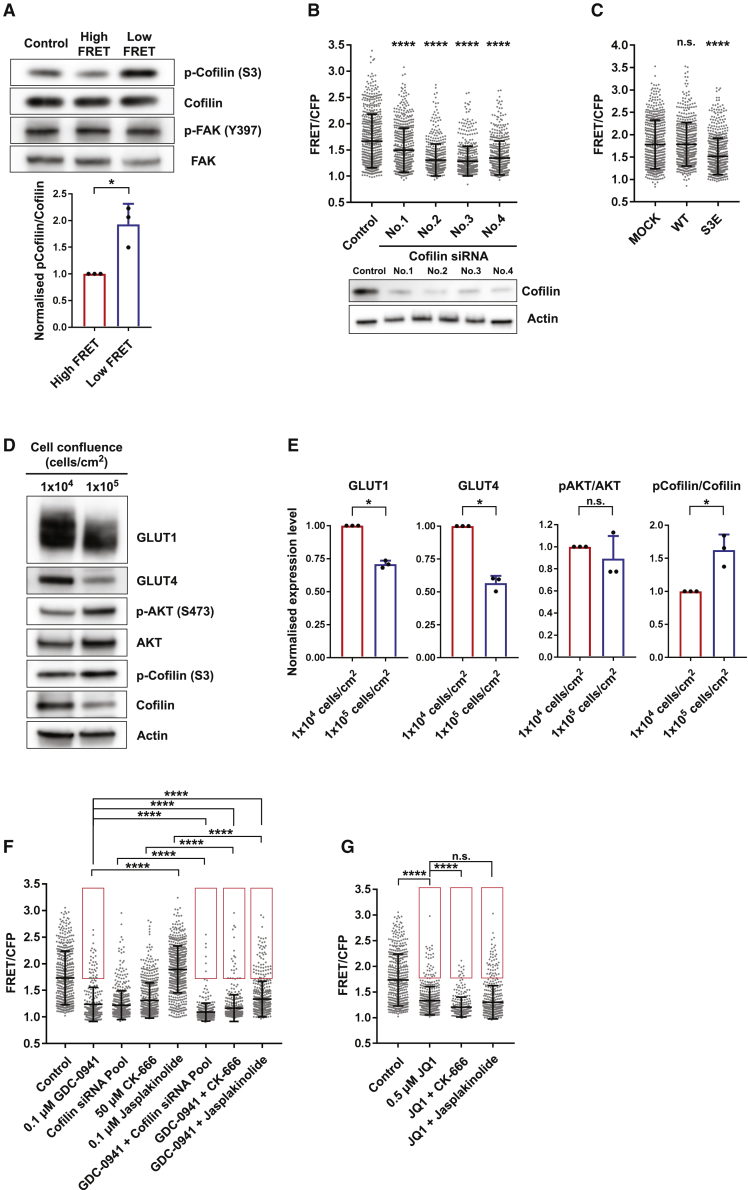
Confluence-dependent control of cofilin activity is a parallel regulator of intracellular glucose concentration (A) Western blot of actin cytoskeletal regulators in FACS-isolated high- and low-glucose-concentration cells. Expression and phosphorylation levels of cofilin were quantified from 3 independent blots. (B) FRET distribution of intracellular glucose levels of MCF-7 cells with cofilin knockdown. n > 450 cells from 3 independent experiments. (C) FRET distribution of intracellular glucose levels of MCF-7 cells expressing wild-type (WT) cofilin-mCherry or S3E coflilin-mCherry. n > 500 cells from 3 independent experiments. (D and E) Western blot of metabolism regulators in MCF-7 cells at 1.0 × 10^4^ cells/cm^2^ and 1.0 × 10^5^ cells/ cm^2^. Expression and phosphorylation levels of GLUT1, GLUT4, AKT, and cofilin were quantified from 3 independent blots. (F) The effect of inhibitors of actin cytoskeletal dynamics and combination therapy with GDC-0941 on intracellular glucose levels of MCF-7 cells. The cells indicated by the red square represent inhibitor-resistant high-glucose-concentration cells. n > 300 cells from 3 independent experiments. (G) The effect of combination therapy of actin cytoskeletal dynamics and JQ1 on intracellular glucose levels of MCF-7 cells. The cells indicated by the red square represent inhibitor-resistant high-glucose-concentration cells. n > 300 cells from 3 independent experiments. Data are shown as mean ± SD. Statistical significance of WB was examined by two-tailed unpaired t test. Statistical significance of glucose FRET biosensor was examined by Kolmogorov-Smirnov test. p values are indicated by ns, p > 0.05; ^∗^p < 0.05; ^∗∗∗∗^p < 0.0001.

The data described above illustrate the importance of PI3K signaling in maintaining cells in the high-glucose state. Given our previous observation of the influence of cell confluence on glucose concentration, we also investigated the activation state of various cytoskeletal regulators that have been linked to changing cell density in cells with differing glycolytic states (Aragona et al., 2013; Demircioglu et al., 2020). Flow sorting was used to separate cells with high- and low-glucose FRET signals. Figure 5A shows that the activity of FAK was equivalent between the high- and low-glucose cells. However, we noted a difference in the phosphorylation of the actin-severing protein cofilin, with higher pS3-cofilin levels in the low-glucose-concentration cells. Phosphorylation of cofilin reduces its ability to sever actin filaments; therefore, we asked if cofilin depletion would be sufficient to drive cells into a low-glucose state. Figure 5B shows that cofilin depletion in sub-confluent cells is sufficient to reduce intracellular glucose levels. Expression of the phospho-mimetic cofilin S3E mutant, but not the wild type, reduced the glucose FRET signal, thereby confirming the importance of cofilin phosphorylation for modulating glucose levels (Figure 5C). Figures 2I and 2J had indicated that high cell confluence reduces intracellular glucose concentration. Interestingly, high cell density led to increased cofilin phosphorylation, which is consistent with cofilin being a regulatory intermediate between cell density and glucose metabolism (Figures 5D and 5E). Furthermore, GLUT1 and GLUT4 levels were also reduced at high cell density, whereas pS473-AKT levels were unchanged. Cofilin depletion did not affect pS473-AKT, and PI3K inhibition did not reduce cofilin activity, as inferred by pS3-cofilin (Figure S5A). These data argue that downregulation of intracellular glucose levels in confluent cells is independent of PI3K activity but linked to reduced cofilin activity and altered glucose transporter levels.

Having established that cofilin is involved in regulation of glucose metabolism, we further tested whether other actin perturbations also influence the glucose FRET signal. Numerous studies have reported cooperativity between cofilin-mediated actin severing and actin nucleation by the Arp2/3 complex in driving cell protrusion and migration (Bravo-Cordero et al., 2013). Inhibition of the Arp2/3 complex using CK-666 rapidly reduced the glucose FRET signal (Figures 5F and S5B). Converse results were obtained with jasplakinolide, which stabilizes F-actin (Figure 5F).

We next investigated the interplay between PI3K- and actin-dependent regulation of the glucose FRET signal. Analysis of PI3K inhibition at different levels of cell confluence revealed that, as confluence increased, the relative efficacy of PI3K inhibition fell. This finding indicates that there is a sub-population of high-glucose FRET cells that are not regulated by PI3K or actin dynamics. Consistent with this result, the combination of PI3K inhibitor treatment and cofilin knockdown or Arp2/3 complex inhibition displayed weakly additive effects, with some refractory cells always being present (Figure 5F). In contrast, bromodomain and Arp2/3 inhibition synergized effectively regardless of confluence (Figures 5F, 5G, and S4F–SH). Jasplakinolide partially rescued the impact of PI3K inhibition on glucose levels (Figure 5F). In contrast, jasplakinolide did not boost the glucose FRET signal when combined with JQ1 (Figure 5G). Together, these data argue that changes in actin dynamics underpin the transition to a low-glucose state upon confluence and establish a regulatory link between cofilin function and intracellular glucose concentration.

### Intravital imaging reveals regional variation in metabolic state

**Figure 6:**
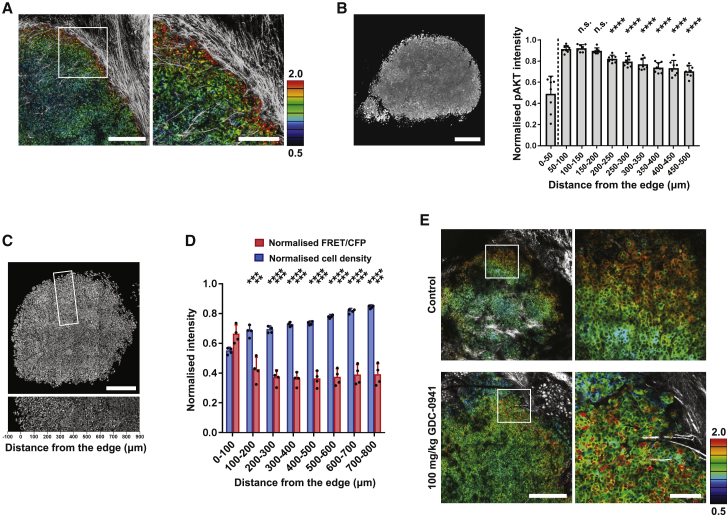
Intravital imaging reveals regional variation in metabolic state (A) Intravital imaging of intracellular glucose levels of a representative MCF-7 tumor in NOD SCID mouse. FRET ratio of glucose FRET biosensor and SHG are displayed in 8-color and gray, respectively. Scale, 500 μm. Scale of high-magnification image, 200 μm. (B) Phosphorylated AKT of resected tumors were immune-stained and quantified in the central-peripheral axis every 50 μm. Scale, 500 μm. (C) Nuclei density in resected MCF-7 tumor in NOD SCID mouse. Nuclei were stained by DAPI. Scale, 500 μm. (D) Intracellular glucose levels and cell density were quantified in the central-peripheral axis every 100 μm. (E) Intravital imaging of intracellular glucose levels of MCF-7 tumor in NOD SCID mice were acquired after 3-day OG treatment of control solution (10% DMSO, 5% Tween-20 in distilled water [D.W.]) or GDC-0941 (100 mg/kg GDC-0941 in 10% DMSO, 5% Tween-20 in D.W.). FRET ratio of glucose biosensor and SHG are displayed in 8-color and gray, respectively. Scale, 500 μm. Scale of high-magnification image, 200 μm. Data are shown as mean ± SD. Statistical significance was examined by two-tailed unpaired t test. p values are indicated by ns, p > 0.05; ^∗^p < 0.05; ^∗∗^p < 0.01; ^∗∗∗^p < 0.001; ^∗∗∗∗^p < 0.0001.

The data above describe how breast cancer cells can transit between metabolic states through the combination of PI3K signaling, actin dynamics, and a permissive bromodomain-dependent chromatin state to drive cancer cells into a highly glycolytic state. We next sought to identify pathways and factors that would determine which state cells adopt *in vivo*. We observed significant cellular heterogeneity within MCF-7 tumors grown in mice by using intravital imaging, and cancer cells at the edge of the tumor had higher levels of intracellular glucose (Figure 6A). The spatial patterns observed by ratiometric FRET were consistent with those observed by fluorescence lifetime measurements of the glucose biosensor, indicating that ratiometric FRET imaging is reliable in this context (Figure S6A). Our analysis of cells with high and low glucose levels suggested that elevated PI3K signaling and lower density might be linked with high intracellular glucose. We therefore interrogated these variables in MCF-7 tumors. Consistent with our findings that glucose levels are higher at the tumor borders, we observed high PI3K signaling, as inferred by pS473-AKT in these regions (Figures 6B and S6B). Analysis of cancer cell density in tumors confirmed the inverse relationship between this parameter and intracellular glucose *in vivo* (Figures 6C, 6D, and S6C). We also investigated whether proximity to blood vessels might account for the variation in the glucose biosensor signal. Figure S6D shows that proximity to a vessel was not sufficient to generate high-glucose FRET signal, and we did not observe any relationship between blood vessel location and distance from the tumor edge (Figure S6E). To directly test whether the PI3K pathway regulates glucose levels *in vivo*, we treated mice with GDC-0941 and observed lower glucose levels in regions immediately adjacent to tumor vasculature in the tumor boundary compared with tumors in DMSO-treated mice (Figure 6E). Intriguingly, refractory cells that retained high intracellular glucose were found widely distributed in GDC-0941-treated tumors. These data demonstrate regional heterogeneity in intracellular glucose levels *in vivo* and support our *in vitro* analysis indicating that PI3K and cell density may be key regulators.

### “Scratch” assays recapitulate the regional variation in metabolic state

**Figure 7:**
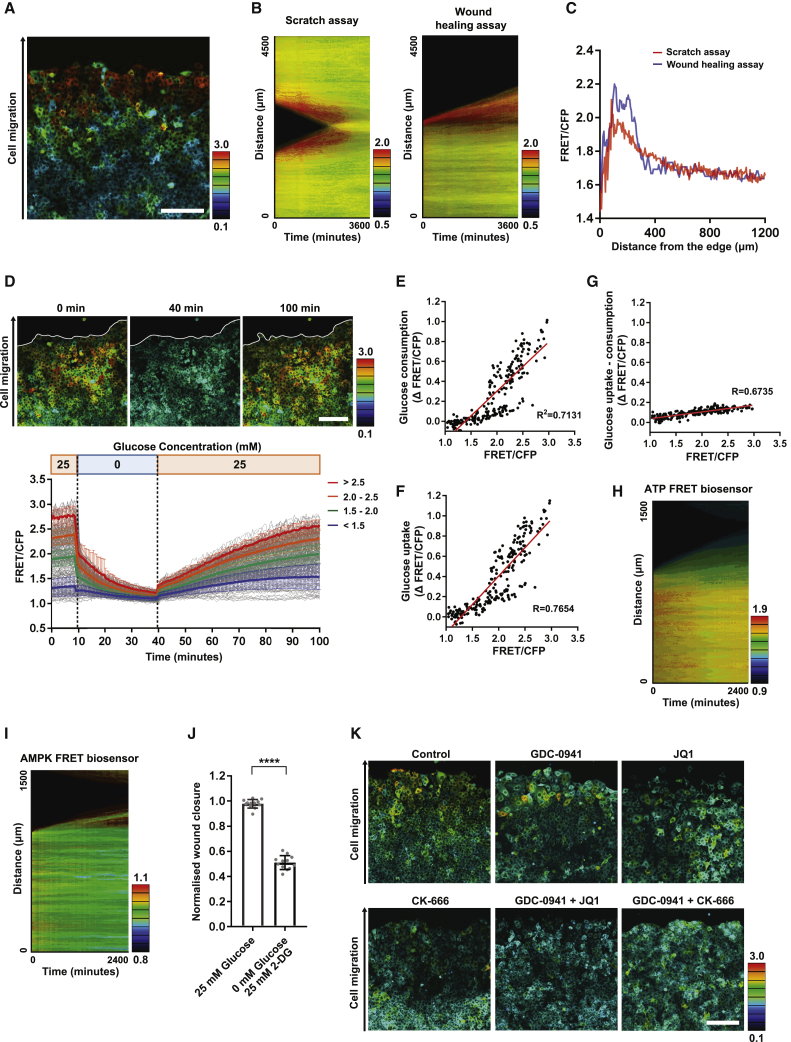
“Scratch” assays recapitulate the regional variation in metabolic state (A) Scratch assay of glucose-FRET-biosensor-expressing MCF-7 cells. FRET signals of glucose FRET biosensor in MCF-7 cells were acquired 24 h after cell scratch. Scale, 100 μm. (B) Kymograph of FRET signals in scratch and wound-healing assay. (C) Quantification of intracellular glucose level in scratch and wound-healing assay. (D) Glucose uptake and consumption speed of MCF-7 cells after scratching the cells. Scratched MCF-7 cells were incubated with 0 mM glucose media at T = 10 min and then replaced with 25 mM glucose media at 40 min. Images were taken every 30 s. n = 221 cells from 3 independent experiments. Scale, 200 μm. (E–G) Glucose consumption, uptake, and uptake-consumption were calculated from the FRET changes of scratched MCF-7 cells. Plots show single-cell tracing of FRET signals and their FRET shifts. n = 221 cells from 3 independent experiments. (H) Kymograph of scratch assay in ATP-FRET-biosensor-expressing MCF-7 cells. The cells were scratched and time-lapse images were acquired for 40 h. (I) Kymograph of scratch assay in AMPK-FRET-biosensor-expressing MCF-7 cells. The cells were scratched, and time-lapse images were acquired for 40 h. (J) Wound-healing assay of MCF-7 cells cultured in 25 mM glucose media or 0 mM glucose with 25 mM 2-DG media. Wound closure was calculated from the wound area at 0 h and 24 h after cell scratch. n = 12 for each condition from 4 independent experiments. (K) The effect of combinatorial inhibition of PI3K, bBromodomain, and cofilin activities on scratch-induced glucose increase. Glucose-FRET-biosensor-expressing MCF-7 cells were incubated with 0.1 μM GDC-0941, 0.5 μM JQ1, or 50 μM CK-666 for 24 h and then the cells were scratched. FRET signals of the glucose FRET biosensor in MCF-7 cells were acquired 24 h after scratching the cells. Scale, 200 μm. Data are shown as mean ± SD. Statistical significance was examined by two-tailed unpaired t test. p values are indicated by ^∗∗∗∗^p < 0.0001.

Having established correlations between elevated glucose levels, high PI3K/AKT signaling, and lower cell density at the tumor margin, we sought to develop a simple assay that could recreate some these features and allow the transition of cells between high- and low-glucose states to be studied. Scratch assays in which space is generated in a confluent monolayer are widely used to explore the migration of epithelial cells into free space and can enable the transition of cells to lower density. Furthermore, previous studies have documented an increase in PI3K signaling and cofilin regulation under these assay conditions (Kanazawa et al., 2010; Kim et al., 2009). Figures 7A and 7B and Video S3 show that intracellular glucose increases dramatically in scratch and wound-healing assays (the latter simply involve the removal of an inert barrier and so exclude the possibility of cell damage response to scratching), with the emergence of a layer of high-glucose cells extending ~200 μm from the newly created border (Figure 7C). The increase in glucose concentration is evident less than 1 h after the scratch and peaks at around 12 h in cells further from the migrating front. Similar results were observed in ZR-75-1 cells (Figure S7A). When cells have migrated across the gap, the glucose FRET signal returns to baseline within 10 h (Figure 7B; Figure S7B). Glucose wash-out/wash-in analysis indicated high rates of glucose uptake and consumption by cells in this region (Figures 7D–7G; Figure S7C; Video S4). Indeed, glucose uptake rates exceeded those observed under routine cell culture conditions. Despite the increased rate of glucose use, cells at the migrating edge experienced energetic stress, as evidenced by both reduced ATP levels and increased AMPK activity (Figures 7H, 7I, S7D, and S7E) (Chennell et al., 2016; Imamura et al., 2009). This inferred increase in metabolic stress when in migrating cells was supported by using imaging for ROS (Figure S7F). Although the higher ROS levels might suggest increased mitochondrial function, the mitochondrial membrane potential observed in high-glucose cells near the cell border did not differ from low-glucose cells (Figure S7G). These data suggest that migrating cells near the scratch edge are under metabolic stress and in a somewhat different state from the high-glucose cells observed under sub-confluent conditions. The importance of glucose metabolism for wound closure was confirmed by using media lacking glucose and use of 2DG, which prevents the utilization of intracellular glucose (Figure 7J). Together, these data suggest that the upregulation of glucose utilization following the generation of free space only partly satisfies the energy demands of cells that are simultaneously migrating and proliferating, and this is required for cells to cope with the increased energetic demands of cell migration.

Video S3. Imaging glucose biosensor signal in scratch assay, related to Figures 7A and 7B Scratch assay of glucose FRET biosensor-expressing MCF-7 cells. Glucose FRET biosensor-expressing MCF-7 cells was imaged for 60 hours every 5 minutes. Scale = 500 μm.

Video S4. Single-cell measurements of the glucose biosensor signal throughout glucose wash-out/wash-in analysis in scratch assay, related to Figures 7D–7G Scratched glucose FRET biosensor-expressing MCF-7 cells were incubated with 25 mM glucose media from 0 to 10 minutes. The media was replaced with 0 mM glucose media at 10 minutes and the cells were incubated for 30 minutes. The scratched cells were re-cultured in 25 mM glucose media from 40 minutes. Images were taken every 30 s. Scale = 100 μm.

Finally, we performed interventional experiments with the aim to explain a hierarchy of regulatory pathways of metabolic transitions. Based on our earlier experiments, we investigated the effect of the PI3K inhibitor, bromodomain inhibition, and targeting actin dynamics using CK666 in the context of scratch assay. Pre-treatment of cells with the PI3K inhibitor reduced the transition to a high-glucose state; however, a subset of cells near the invading front remained refractory to PI3K inhibition (Figure 7K). Similar to our observations in sub-confluent cultures, these cells could be effectively targeted by combining PI3K inhibition with bromodomain inhibition. Co-inhibition of PI3K and Arp2/3 function did not further deplete the high-glucose population at the migrating front. Together, these data reveal extensive inter-cellular heterogeneity in glucose use in breast cancer, both *in vitro* and *in vivo*. Furthermore, they reveal how cell density and cell migration interact with PI3K signaling and bromodomain functionality to drive cells into high-glycolysis states.

## Discussion

In this work, we uncover how inter-cellular heterogeneity in metabolic state in breast cancer results from the combined variation in PI3K signaling, chromatin state, and actin dynamics (Figures S7H and S7I). Fluorescent biosensors have previously been used to perform high-content screens for metabolic perturbations, as well as interrogate metabolic features *in vivo*; however, one under-explored feature that these tools provide is the ability to perform longitudinal single-cell analysis (Takanaga et al., 2008; Zhao et al., 2015; Zou et al., 2020). Previous work using multiplex imaging of an AKT biosensor and AMPK FRET biosensor has shown that the cellular energetic state is synchronized with AKT activity (Hung et al., 2017). By multiplexing FRET imaging with traditional readouts of metabolism, we have generated multiparametric insights into heterogeneity in cell state and its plasticity within breast cancer cells cultured under uniform conditions. We demonstrate that metabolic state is a heritable trait. The basis for heritability is, most likely, epigenetic regulation, with bromodomain readers playing a key role. Previous work has shown that bromodomain function synergizes with PI3K activity to regulate glycolysis (Stratikopoulos et al., 2015). However, additional analysis is required to determine if this mechanism is relevant in our system. Our data simply argue that a subset of breast cancer cells with high bromodomain function are completely refractory to inhibition of PI3K signaling (top left in Figure S7H and also in Figure S7I). Thus, combined targeting of PI3K and bromodomain function has two benefits: it reduces the expression of key PI3K regulators and it targets a sub-population of cells with high glycolysis driven by a non-PI3K mechanism. This latter mechanism may also account for the modest difference in PI3K activity between the high- and low-glucose FRET cells. As might be expected for epigenetic regulation, the temporal linkage between chemical perturbation and changing intracellular glucose levels exhibit a long delay—in the range of 24 h. In contrast, perturbation of PI3K activity and actin dynamics affect intracellular glucose within 30 min.

Actin dynamics are crucially dependent on ATP, both for the actin treadmilling cycle and for myosin function (Pollard, 2016). It is increasingly appreciated that actin dynamics also provide regulatory input in various steps in glycolysis (Hu et al., 2016; Papalazarou et al., 2020; Park et al., 2020). Furthermore, physical barriers shift metabolic requirements as cancer cells generate ATP to shape and breakdown extracellular matrix (Zanotelli et al., 2018, 2019; Zhang et al., 2019). In this work, we show that cofilin activity is crucial for high intracellular glucose levels. Furthermore, cofilin activity is reduced as cells become confluent and intracellular glucose levels fall. Relieving the restriction of high confluence by creating a scratch or wound *in vitro* triggers a transition to a high-glucose state with high-glucose uptake rates. The mechanisms for this upregulation are unclear but could include increased PI3K signaling at the migrating front promoting GLUT1 and GLUT4 trafficking to the plasma membrane or simply the larger membrane area of the migrating cells favoring glucose uptake. In the future, it will be interesting to investigate these possibilities further. In this study, we observe that edges in epithelial wound assays as well as tumor boundaries are sites of metabolic transitions with both elevated PI3K signaling and lower cancer cell density favoring a high-glucose state (right hand in Figure S7H). Similar to the situation *in vivo*, inhibition of PI3K signaling in wound assays leads to fewer and more disparately distributed high-glucose cells. In this subset of cells, high glucose levels are driven by bromodomain function (Figure S7I). The combination of PI3K and bromodomain inhibition effectively targets all cancer cells. Interestingly, combining PI3K inhibition with actin perturbation provides relatively little advantage over targeting either mechanism alone. Although our data do not support the view that PI3K is upstream of cofilin regulation or vice versa, these data can be explained if both PI3K signaling and cofilin function both impinge on the same regulatory step in glycolysis. Together, our data and those of others support potential mechanisms of convergence, including GLUT1 and FAK, but also the localization and stability of aldolase A and phosphofructokinase, respectively (Hu et al., 2016; Park et al., 2020). Despite the actin-dependent increase in glucose uptake in migrating cells, the cells at the wound edge still experience energetic stress with lower ATP levels and increased AMPK activity. Therefore, the increase in glucose uptake cannot fully compensate for the increased energy demands of the migrating cells.

Cancer cells in distinct states are likely to have differing vulnerabilities. Indeed, we observe that high-glucose cells are particularly depending on extra-cellular pyruvate. However, this vulnerability is masked in the presence of either low-glucose cancer cells or stromal fibroblasts. Thus, it will be important to understand non-cell-autonomous mechanisms of metabolic crosstalk, as well as consider inter-cellular heterogeneity if therapies are to optimized (Kreuzaler et al., 2020; McGuirk et al., 2020). The tools and approaches described here should be valuable assets in this endeavor.

## STAR★methods

### Key Resources Table

**Table:** 

REAGENT or RESOURCE	SOURCE	IDENTIFIER
**Antibodies**		

GLUT1	Abcam	Cat # ab15309; RRID:AB_301844
GLUT4	Abcam	Cat # ab654; RRID:AB_305554
phospho AKT (Ser473)	Cell Signaling	Cat # 4060; RRID:AB_2315049
AKT	Cell Signaling	Cat # 2920; RRID:AB_1147620
Phospho S6 Ribosomal Protein (Ser235/236)	NEB	Cat # 2211S; RRID:AB_331679
S6 Ribosomal Protein	NEB	Cat # 2317
phospho Cofilin (Ser3)	Cell Signaling	Cat # 3311S; RRID:AB_330238
cofilin	Cell Signaling	Cat # 3312; RRID:AB_330235
phospho FAK (Tyr397)	Cell Signaling	Cat # 8556S
FAK	Cell Signaling	Cat # 3285S; RRID:AB_2269034
BRD4	Abcam	Cat # ab128874; RRID:AB_11145462
CD31 Alexa Fluor 594 (Clone WM59)	BioLegend	Cat # 303126; RRID:AB_2563303
GFP	Abcam	Cat # ab13970; RRID:AB_300798
β-Actin	Sigma-Aldrich	Cat # A1978; RRID:AB_476692
Goat anti-Rabbit IgG, Alexa Fluor 555	Thermofisher Scientific	Cat # A-21429
Goat anti-Mouse IgG (H+L) Secondary Antibody, HRP	Thermofisher Scientific	Cat # 31430
Goat anti-Rabbit IgG (H+L) Secondary Antibody, HRP	Thermofisher Scientific	Cat # 31460

**Chemicals, peptides, and recombinant proteins**		

Oligomycin A	Tocris Bioscience	Cat # 4110/5
Koningic acid, GAPDH inhibitor	Abcam	Cat # ab144269
FCCP, Proton uncoupler of mitochondria	Sigma-Aldrich	Cat # C2920
Rotenone, Mitochondrial Complex 1 inhibitor	Bio-techne	Cat # 3616/50
Antimycin A, Mitochondrial Complex 3 inhibitor	Sigma-Aldrich	Cat # A8674
Pictilisib (GDC-0941), PI3Kα/δ inhibitor	Selleckchem	Cat # S1065
Alpelisib (BYL719), PI3Kα inhibitor	Selleckchem	Cat # S2814
AZD8186, PI3Kδ/β inhibitor	Cambridge Bioscience	Cat # 17384
JQ1, BET bromodomain inhibitor	Selleckchem	Cat # S7110
AZD5153, BRD4 inhibitor	Selleckchem	Cat # S8344
CK-666, Arp2/3 inhibitor	Tocris Bioscience	Cat # 3950/10
Jasplakinolide, Actin de-polymerization inhibitor	Tocris Bioscience	Cat # 2792/100
2-DG	Sigma-Aldrich	Cat # D8375
TMRE	Thermofisher Scientific	Cat # T669
CellROX Deep Red Reagent	Thermofisher Scientific	Cat # C10422
2-NBDG	Thermofisher Scientific	Cat # N13195
Tetramethylrhodamine isothiocyanate-Dextran	MERCK	Cat # T1287
17β-ESTRADIOL (0.72mg/pellet, 90 day release)	Innovative Research of America	Cat # NE-121

**Critical commercial assays**		

Seahorse XFe96 FluxPak mini	Agilent	Cat # 102601-100

**Experimental models: cell lines**		

Human: MCF-7 cells	Cell Servies of The Francis Crick Institute	N/A
Human: T47D cells	Cell Servies of The Francis Crick Institute	N/A
Human: ZR-75-1 cells	Cell Servies of The Francis Crick Institute	N/A
Human: MDA-MB-231 cells	Cell Servies of The Francis Crick Institute	N/A
Human: 1902T CAFs cells	Louise Jones’s laboratory in CRUK Barts Centre	N/A

**Experimental models: organisms/strains**		

Mouse: NOD.Cg-*Prkdc*^*scid*^/J	The Jackson Laboratory	Stock No: 001303

**Oligonucleotides**		

siRNA targeting sequence: BRD4 #1:	Dharmacon	Cat # J-004937-06-0002
siRNA targeting sequence: BRD4 #2:	Dharmacon	Cat # J-004937-08-0002
siRNA targeting sequence: BRD4 #3:	Dharmacon	Cat # J-004937-09-0002
siRNA targeting sequence: Cofilin #1:	Dharmacon	Cat # D-012707-01
siRNA targeting sequence: Cofilin #2:	Dharmacon	Cat # D-012707-02
siRNA targeting sequence: Cofilin #3:	Dharmacon	Cat # D-012707-03
siRNA targeting sequence: Cofilin #4:	Dharmacon	Cat # D-012707-04

**Recombinant DNA**		

pcDNA3.1 FLII12Pglu-700uDelta6	(Takanaga et al., 2008)	Addgene Plasmid #21339
ATeam1.03-nD/nA/pcDNA3	(Kotera et al., 2010)	Addgene Plasmid #21339
T2AMPKAR-NES	David Carling and Alessandro Sardini’s laboratory, Imperial College London	N/A
pSBbi-FoxO1_1R_10A_3D	(Gross et al., 2019)	Addgene Plasmid #106278

**Software and algorithms**		

ImageJ	Wayne Rasband	https://imagej.nih.gov/ij/
MetaMorph	Molecular Devices	https://www.moleculardevices.com/products/cellular-imaging-systems/acquisition-and-analysis-software/metamorph-microscopy#gref

### Resource availability

#### Lead contact

Further information on resources and reagents should be directed to Lead Contact, Erik Sahai (Erik.Sahai@crick.ac.uk).

#### Materials availability

Plasmids generated in this study will be submitted to Addgene.

#### Data and code availability

No custom code was used in this study.

### Experimental model and subject details

#### Cell culture and transfection

Human breast cancer cell lines, T47D, ZR-75-1, MCF-7, MDA-MB-231 cells were cultured in Dulbecco’s modified Eagle medium (DMEM) with 10% fetal bovine serum (FBS) and 1% Penicillin Streptomycin (Pen Strep) at 37°C in 5% CO_2_. The Francis Crick Institute Cell Services screened these cell lines for mycoplasma. Before experiments, the cells were detached from cell culture dishes by 0.1% trypsin (Thermo Fisher Scientific) and 0.02% versene solution (Thermo Fisher Scientific). Cells were plated on glass bottom dishes at 1 × 10^4^ cells / cm^2^ and media was changed to phenol red-free DMEM with 10% FBS at 3 hours before imaging. For ratiometric FRET and FLIM-FRET imaging, FRET biosensor stably-expressing breast cancer cell lines were made using the PiggyBac transposon system. DNA plasmids were transfected using Lipofectamine 2000 reagent (Thermo Fisher Scientific). After selection of FRET biosensor-expressing cells using puromycin, FRET biosensor-expressing cells were sorted by BD fluorescence-activated cell sorting (FACS) Aria III. The cells were excited by 405 nm and 488 nm for cyan and yellow fluorescent proteins. Fluorescence of these two fluorescent proteins was detected using 450/50 nm and 525/50 nm band pass filters.

#### Mice

The study is compliant with all relevant ethical regulations regarding animal research. All protocols were in accordance with UK Home Office regulations under project license PPL70/8380 and subsequently PP0736231, which passed ethical review by the Francis Crick Institute Animal Welfare Ethical Review Board in 2019. Within the Biological Research Facility (BRF) animal units of the Francis Crick institute, mice had *ad libitum* access to feed and water; and were housed in individually ventilated cages maintained at 22°C with 60% humidity on a 12 hour light-dark cycles. Female mice at 6-12 weeks old were used for all mouse experiments.

### Method details

#### Plasmids

FRET biosensors in the PiggyBac transposon vector were cloned from the Glucose FRET biosensor (Addgene, 17866) and the ATP FRET biosensor (Addgene, 51958). The AMPK FRET biosensor was gifted from David Carling and Alessandro Sardini’s laboratory, Imperial College London. The D183A point mutation in the glucose FRET biosensor was generated by a site-directed mutagenesis kit (NEB). The glucose FRET biosensor with mTurquoise2 and sReACh combination was constructed using Gibson Assembly (NEB) and cloned in a PiggyBac transposon vector. The QIAfilter Plasmid Maxi kit (QIAGEN) was used for plasmid DNA purification and DNA concentration was measured by the Nanodrop Spectrophotometer (Nanodrop) at 260 nm absorbance.

#### Western Blot

Protein expression levels were measured by western blot. MCF-7 cells were grown to roughly 80% confluence in 60 mm dishes and lysed with ice-cold lysis buffer (50 mM HEPES pH 7.4, 150 mM NaCl, 5% Glycerol, 1% NP-40, Proteinase inhibitor tablet, Phosphatase inhibitor tablet). Cell lysates were mixed with 4 x SDS sample buffer (180 mM HEPES pH 7.4, 40% glycerol, 4% SDS, 4% beta-mercaptoethanol, 0.04% bromophenol blue) and heated at 95°C for 5 minutes. Next, these samples were loaded to 4%–15% Mini-PROTEAN® TGX precast gels (Bio-Rad) along with a prestained protein ladder (Benchmark, Invitrogen) for electrophoresis. Proteins were then transferred to 0.2 μm Trans-Blot® Turbo Mini PVDF membrane (Bio-Rad). The membrane was blocked with 5% BSA in Tris buffer saline containing 0.1% Tween-20 for 1 hour at room temperature and incubated with primary antibodies overnight at 4°C. The membrane was next incubated with Horseradish peroxidase (HRP)-coupled secondary antibodies for 1 hour at room temperature. Protein bands were revealed by Immobilon Luminata Crescendo Western HRP substrate (EMD Millipore) and chemiluminescence signals were detected by Amersham Imager 600 (GE Healthcare Life Sciences). Protein band intensity was measured and quantified by ImageJ software.

#### *In vitro* imaging

MCF-7 cells were plated on glass bottom dishes at 1 × 10^4^ cells / cm^2^. For inhibitor treatments, the inhibitors were diluted in DMEM with 10% FBS and cells were incubated with inhibitors for 24 hours at 37°C in 5% CO_2_. For gene knockdown, siRNA was transfected using DharmaFECT 2 reagent (Dharmacon) and incubated for 48 hours at 37°C in 5% CO_2_. 3 hours before imaging, the media was changed to phenol red free DMEM with 10% FBS. Images of the FRET biosensor were acquired using an inverted Zeiss LSM 780 confocal microscope. FRET images of the glucose FRET biosensor were acquired with 458 nm excitation from the argon ion laser. Excitation light from the argon ion laser passed through a MBS 458 dichroic beam splitter. The cells were imaged using Plan-Apochromat 20x/0.8 NA objective lens and the emission signals were detected using GaAsP detectors. For imaging the glucose and ATP FRET biosensors, fluorescence was separated by beam splitters into 464-508 nm for eCFP and a 516-550 nm for sensitized emission (FRET). For imaging the AMPK FRET biosensor, fluorescence was separated by beam splitters into 464-499 nm for mTFP and a 516-550 nm for sensitized emission (FRET). The emission signals of two channels were simultaneously acquired with 458 nm excitation. For imaging 2-NBDG, fluorescence was separated by beam splitter into 516-560 nm. The fluorescence of TMRE and CellRox Deep Red were separated by beam splitters into 590 - 630 nm and 635 - 691 nm, respectively.

#### *In vivo* imaging

Mouse xenograft tumors of MCF-7 cells were prepared by mammary fat pad injection in female NOD SCID mice at 6-12 weeks old. Beta-estradiol pellets (0.72mg/pellet, 90 day release, Innovative Research of America) were implanted in NOD SCID mice a week before cell injection in order to provide estrogen required by MCF-7 cells for proliferation. After a week of pellet implantation, glucose FRET biosensor-expressing MCF-7 cells were trypsinized and re-suspended in 20% Matrigel (2 mg / ml) and 20% collagen I (2 mg / ml) in PBS at 2 × 10^7^ cells / ml. 2 × 10^6^ cells were injected under the fourth nipple into the mammary fat pad in Beta-estradiol pellet-implanted NOD SCID mice. 10 - 14 days after cell injection, tumor size reached around 3 - 4 mm and images of developed tumors were acquired under terminal anesthesia using isofluorane.

Intravital images of the glucose FRET biosensor were acquired using an inverted Zeiss LSM 780 confocal microscope. Mice having 3-4 mm sized tumors underwent intravital imaging under terminal anesthesia using isofluorane. A small incision was made in the skin to expose the tumor surface. The mouse was positioned on a heated microscope stage and the tumor surface was placed on the cover glass. The mouse was anaesthetized throughout the imaging session. FRET images of the glucose FRET biosensor were acquired with 850 nm excitation from a Mai Tai laser (Spectra-Physics). Excitation light from the Mai Tai laser passed through a MBS +690 dichroic beam splitter. Tumors were imaged using Plan-Apochromat 10x/0.45 NA or Plan-Apochromat 20x/0.8 NA objective lens and the emission signals were passed through a full opened pinhole (600 μm) and detected in two channels simultaneously using two GaAsP detectors. Fluorescence was separated by beam splitters into 464-508 nm for eCFP and a 516-550 nm for sensitized emission (FRET). The second harmonic generation (SHG) signal was acquired with 850 nm excitation and 390-450 nm signal detection. SHG images were sequentially acquired following with glucose FRET biosensor image acquisition.

Immunohistochemical stains were performed on 5 μm tissue sections from xenograft tumors. Xenograft tumors were quickly resected from the mice after intravital imaging and fixed with 4% PFA at 4°C overnight. Fixed tumors were incubated with 30% sucrose in PBS at 4°C until tumors sank to the bottom of the tube. The tumors were then immersed in OCT (Thermo Fisher Scientific) and frozen by ethanol chilled on dry ice. OCT blocks were sliced in 5 μm sections using a CM3050s Cryostat (Leica). The slices were permeabilized with 0.1% Triton X-100 in PBS for 5 minutes and blocked with 5% BSA in PBS for 1 hour at 4°C. The sections were incubated with primary antibodies at 4°C overnight and then incubated with Alexa Fluor dye-coupled secondary antibodies for 1 hour at room temperature. Nuclei were stained with DAPI.

#### Fluorescence lifetime imaging

A Simple-Tau TCSPC system (Becker & Hickl) with SPC-150 modules was connected to a Zeiss LSM 780. A Hybrid GaAsP detector was put in place for signal detection. Excitation light from the Mai Tai laser (90 MHz) passed through a 690 nm dichroic and a 690 nm short pass emission filter was employed to block excitation light in the detection channel. eCFP or mTurquoise2 fluorescence from the glucose FRET biosensor were acquired with 850 nm excitation. Fluorescence was collected using a 465-495 nm band pass filter. Fluorescence decays were measured using multifunctional 64 bit Data Acquisition Software. Images of 256 × 256 pixels with 256 time bins were acquired with 60 s exposure in order to acquire ~1000 photons per pixel. In order to accurately calculate the fluorescence lifetime, the instrument response function (IRF) was determined by imaging a sample consisting of gold nanorods (Sigma Aldrich) (Talbot et al., 2011), which were measured under identical imaging conditions as the FRET biosensor.

#### Cell proliferation assay

*For IncuCyte S3* Live-Cell analysis, MCF-7 cells were plated on 96 well plates at 1 × 10^4^ cells / cm^2^. The cells were incubated in DMEM with 10% FBS and the plates were placed into an *IncuCyte S3 inside an incubator. Phase contrast images of the cells were acquired every 3 hours for 7 days using 10x objective. The average cell confluence was calculated from 3 phase contrast images acquired in each well. The growth rate of the cells was calculated as the change in cell confluence over time.*

#### Seahorse assay

One day before oxygen consumption rate (OCR) measurement, the Seahorse XFe96 Analyzer (Agilent) was turned on and warmed up to 37°C. MCF-7 cells were plated on XF96 Cell Culture Microplates at 1 × 10^4^ cells / well and cultured in DMEM with 10% FBS at 37°C. XFe96 Sensor Cartridges were hydrated by calibrant solution at 37°C in a non-CO_2_ incubator overnight. For OCR measurements, culture media was changed to Seahorse XF media (1 mM Pyruvate, 2 mM Glutamine, 25 mM Glucose, pH7.4) and incubated at 37°C in the absence of CO_2_ for 1 hour. OCR was measured 3 times after drug treatments with 6 minute intervals. Oligomycin, FCCP, and AntimycinA / Rotenone were added to final concentrations of 1 μM, 0.5 μM, and 0.5 μM respectively to measure “basal respiration,” “ATP production,” and “maximal respiration.”

#### Metabolomics

Metabolite profiling of MCF-7 cells under different conditions was performed by gas chromatography-mass spectrometry (GC-MS) analysis. For the metabolomic analysis of high and low glucose concentration MCF-7 cells, FACS-isolated high and low glucose concentration cells were plated on 60 mm dishes at 5 × 10^5^ cells / dish and cultured in DMEM with 10% dialyzed FBS (Sigma) overnight at 37°C. For stable isotope labeling, cells were cultured in glucose-free DMEM with 10% dialyzed FBS and 25 mM U-^13^C_6_-glucose (CK isotope) for 90 minutes at 37°C. The cells were washed with ice-cold PBS and suspended with 1.5 mL of metabolite extraction solution (water: methanol: chloroform = 1:3:1). The cell suspension was then sonicated and centrifuged at 13,000 x g for 15 minutes. The supernatant was dried in a Speed Vac vacuum concentrator (Christ) and re-suspended in 50 μL chloroform, 150 μL methanol, and 150 μL water. The upper aqueous phase, containing polar metabolites, was dried and twice washed with methanol. Data acquisition was performed largely as previously described (MacRae et al., 2013), using an Agilent 7890A-5975C GC-MS in EI mode after derivatization of dried extracts by addition of 20 μL methoxyamine hydrochloride (20 mg/mL in pyridine (both Sigma), RT, > 16 hours) and 20 μL BSTFA + 1% TMCS (Sigma, RT, > 1 hour). GC-MS parameters were as follows: carrier gas, helium; flow rate, 0.9 ml/minute; column, DB-5MS (Agilent); inlet, 270°C; temperature gradient, 70°C (2 minutes), ramp to 295°C (12.5°C/minute), ramp to 320°C (25°C/minute, 3 minutes hold). Scan range was m/z 50-550. Data was acquired using MassHunter software (version B.07.02.1938). Data analysis was performed using MANIC software, an in house-developed adaptation of the GAVIN package (Behrends et al., 2011). Metabolites were identified and quantified by comparison to authentic standards, and label incorporation estimated as the percentage of the metabolite pool containing one or more ^13^C atoms after correction for natural abundance.

#### Migration Assays

In scratch assay, MCF-7 cells were plated on glass bottom dishes at 1 × 10^5^ cells / cm^2^ and cultured in phenol red free DMEM with 10% FBS for 24 hours at 37°C. The cells were scratched at the center of the well and time-lapse images were acquired every 5 minutes using an inverted Zeiss LSM 780 confocal microscope. In wound healing assay, the removable culture inserts (Ibidi) were placed on glass bottom dishes and MCF-7 cells were plated at 2 × 10^4^ cells / insert. Cells were cultured in phenol red free DMEM with 10% FBS for 24 hours at 37°C. The removable culture inserts were removed from the glass bottom dishes and time-lapse images were acquired every 5 minutes using an inverted Zeiss LSM 780 confocal microscope. Cells were imaged using a Plan-Apochromat 20x/0.8 NA objective lens and the emission signals were detected using GaAsP detectors. Wound closures and FRET ratio were measured and quantified by ImageJ software.

### Quantification and statistical analysis

#### Image analysis

In the calculation of FRET ratio, individual cells were segmented and FRET signals were measured by mean pixel intensities of donor and sensitized emission channels. The 8 colors ratiometric images were created using MetaMorph software. The false-color range used to display ratiometric FRET images were manually fixed for each FRET biosensor. For time-lapse single cell analysis, individual cells were manually segmented and the FRET ratio at each time point was calculated. Kymographs were created from time-lapse images of glucose FRET biosensor-expressing breast cancer cells in the scratch and wound healing assay using ImageJ software.

For the measurement of glucose consumption rate, FRET slops of MCF-7 cells cultured in 0 and 25 mM glucose media were estimated from one phase decay fit. The glucose consumption of single cells was calculated from the FRET shifts during 10 - 13 minutes.

The FRET ratio of the glucose FRET biosensor in xenograft tumors was quantified in the center-periphery axis using ImageJ software. Glucose FRET biosensor-expressing MCF-7 tumors were circled by a segmented line with 2,000 pixel line width. The circled tumors were aligned in a straight line and re-sliced. Re-sliced images of donor and acceptor channels were processed to yield sum slice projections. The intensity of acceptor fluorescence intensity was then divided by the intensity donor fluorescence intensity. The FRET ratio of the glucose FRET biosensor of the xenograft tumor was then calculated in the center-periphery axis. The FRET ratio of glucose FRET biosensor in xenograft tumors was thus quantified from the whole tumor. The quantified FRET ratio of the glucose FRET biosensor represents the average FRET signals of tumors in the center-periphery axis.

Nuclei density, pAKT and CD31 levels of xenograft tumors were quantified in the center-periphery axis using ImageJ software. DAPI, pAKT, and CD31 stained xenograft tumors were circled by a segmented line with 2,000 pixel line width. The circled tumors were aligned in a straight line and re-sliced. Re-sliced images were processed to yield sum slices projections. The nuclei density, pAKT, and CD31 levels of xenograft tumors was quantified in the center-periphery axis.

The fluorescence lifetime images of the glucose FRET biosensor were calculated using the FLIMfit software (Warren et al., 2013). Raw decay data and the gold nanorod IRF were loaded and smoothed with 3x3 spatial averaging to reduce noise. The fluorescence lifetime was calculated for pixels having an integrated fluorescence signal of greater than 100 photons. The mean fluorescence lifetime of the glucose FRET biosensor was calculated for each pixel from a double exponential decay fit to the data with automatic estimation of the initial guesses for the two decays.

#### Statistics

Statistical analyses were performed using GraphPad Prim. The two-tailed unpaired t test was used for the analysis of experimental groups with a Gaussian distribution and the same variance. The Kolmogorov-Smirnov test was used for the analysis of experimental groups with unmatched groups and unequal variance. The P value is calculated to assess if there is a statistically significant difference between the mean of two groups. Each symbol has the following meanings. ns (p > 0.05), ^∗^(p < 0.05), ^∗∗^(p < 0.01), ^∗∗∗^(p < 0.001), ^∗∗∗∗^(p < 0.0001). The R^2^ value quantifies the fraction of all the variation in the samples that is accounted for by a difference between the group means.

## Supplemental information

Document S1. Figures S1–S7

Document S2. Article plus supplemental information
