# Supplementary material for: Single-cell resolved imaging reveals intra-tumor heterogeneity in glycolysis, transitions between metabolic states, and their regulatory mechanisms
Source: Cell Rep. 2021 Feb 16;34(7):108750. doi: 10.1016/j.celrep.2021.108750 (PMC7900713; doi:10.1016/j.celrep.2021.108750)

Cell Reports, Volume *34*

Supplemental Information

Single-cell resolved imaging reveals intra-tumor heterogeneity in glycolysis, transitions between metabolic states, and their regulatory mechanisms

Hiroshi Kondo, Colin D.H. Ratcliffe, Steven Hooper, James Ellis, James I. MacRae, Marc Hennequart, Christopher W. Dunsby, Kurt I. Anderson, and Erik Sahai

Figure S1

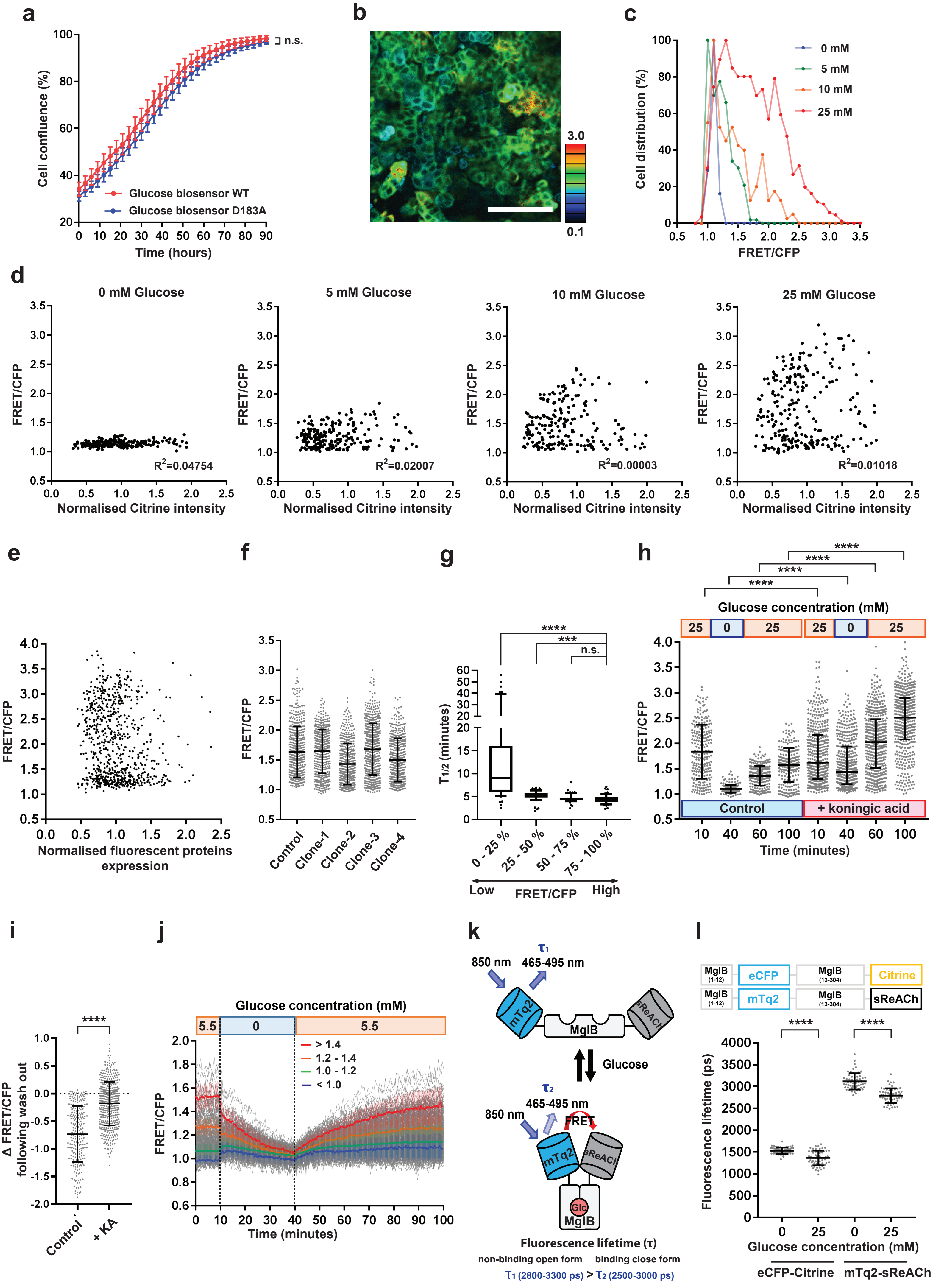

a
Cell confluence (%)
Time (hours)
n.s.
Glucose biosensor WT
Glucose biosensor D183A
b
3.0
0.1
c
Cell distribution (%)
FRET/CFP
0 mM
5 mM
10 mM
25 mM
d
0 mM Glucose
5 mM Glucose
10 mM Glucose
25 mM Glucose
FRET/CFP
Normalised Citrine intensity
R²=0.04754
R²=0.02007
R²=0.00003
R²=0.01018
e
FRET/CFP
Normalised fluorescent proteins expression
f
FRET/CFP
Control
Clone-1
Clone-2
Clone-3
Clone-4
g
T1/2 (minutes)
0 - 25 %
25 - 50 %
50 - 75 %
75 - 100 %
Low
FRET/CFP
High
n.s.
h
Glucose concentration (mM)
25
0
25
25
0
25
FRET/CFP
Control
+ koningic acid
Time (minutes)
i
Δ FRET/CFP following wash out
Control
+ KA
j
Glucose concentration (mM)
5.5
0
5.5
> 1.4
1.2 - 1.4
1.0 - 1.2
< 1.0
FRET/CFP
Time (minutes)
k
850 nm
τ1
465-495 nm
mTq2
MglB
sReACh
Glucose
τ2
465-495 nm
850 nm
FRET
Glc
MglB
Fluorescence lifetime (τ)
non-binding open form
binding close form
τ1 (2800-3300 ps) > τ2 (2500-3000 ps)
l
MglB (1-12)
eCFP
MglB (13-304)
Citrine
MglB (1-12)
mTq2
MglB (13-304)
sReACh
Fluorescence lifetime (ps)
Glucose concentration (mM)
eCFP-Citrine
mTq2-sReACh

Figure S1. Breast cancer cells exhibit heterogeneity in metabolic state even in controlled culture conditions. Figure S1 is related to Figure 1.

(A) Growth of stable MCF7 cells expressing wild type (WT) and D183A glucose FRET biosensor. Cell confluence was measured every 3 hours using IncuCyte *S3* Live-Cell Analysis System. Data are shown as mean ± SD. n = 20 wells from three independent experiments. **(B)** Intracellular glucose levels of a representative MCF-7 tumour in NOD SCID mouse. Scale = 100 µm. **(C)** FRET distribution of glucose FRET biosensor-expressing MCF-7 cells cultured in 0, 5, 10, or 25 mM glucose media for one hour. **(D)** Glucose-dependent FRET signals plotted against normalised Citrine intensity in individual cells cultured in 0, 5, 10, or 25 mM glucose media for one hour in each condition. n > 200 cells from three independent experiments. **(E)** FRET signals of glucose FRET biosensor plotted against normalised GFP intensity in individual cells. Fluorescence proteins in biosensor were stained by anti-GFP antibody. n > 700 cells from three independent experiments. **(F)** Intracellular glucose levels of MCF-7 clones 1 month after single cell isolation. Data are shown as mean ± SD. n > 400 cells in each clone. **(G)** Half-life of intracellular glucose levels. Individual curves were fitted by one phase decay. Data are shown as box plots of median with lower to higher quartiles, and 10 to 90 percentile whiskers. **(H)** Glucose uptake and consumption speed of MCF-7 cells treated with 10 µM koningic acid. Glucose FRET biosensor-expressing MCF-7 cells were incubated with 0 mM glucose media at 10 minutes, and re-cultured with 25 mM glucose media at 40 minutes. Data are shown as mean ± SD. n > 200 cells from three independent experiments. **(I)** FRET shifts of glucose FRET biosensor in MCF-7 cells 30 minutes after incubation in 0 mM glucose media. Data are shown as mean ± SD. n > 200 cells from three independent experiments. **(J)** Glucose uptake and consumption speed of MCF-7 cells cultured in Plasmax media. Glucose FRET biosensor-expressing MCF-7 cells were incubated with 0 mM glucose media at 10 minutes, and re-cultured with 5.5 mM glucose media at 40 minutes. Images were taken every 30 seconds. Data are shown as mean ± SD. n > 200 cells from three independent experiments. **(K)** Schematic representation of the conformational shift of the glucose FRET biosensor with mTurquoise2-sReACh combination. **(L)** Fluorescence lifetime of glucose FRET biosensors with different fluorophore combinations. The cells were incubated in 0 or 25 mM glucose media for 1 hour and then fluorescence lifetime of the donor fluorophore was measured by TCSPC FLIM. Data are shown as mean ± SD. n > 50 cells from three independent experiments Statistical significance was examined by Kolmogorov-Smirnov test. P values are indicated by ns ($p > 0.05$), *** ($p < 0.001$), **** ($p < 0.0001$).

Figure S2

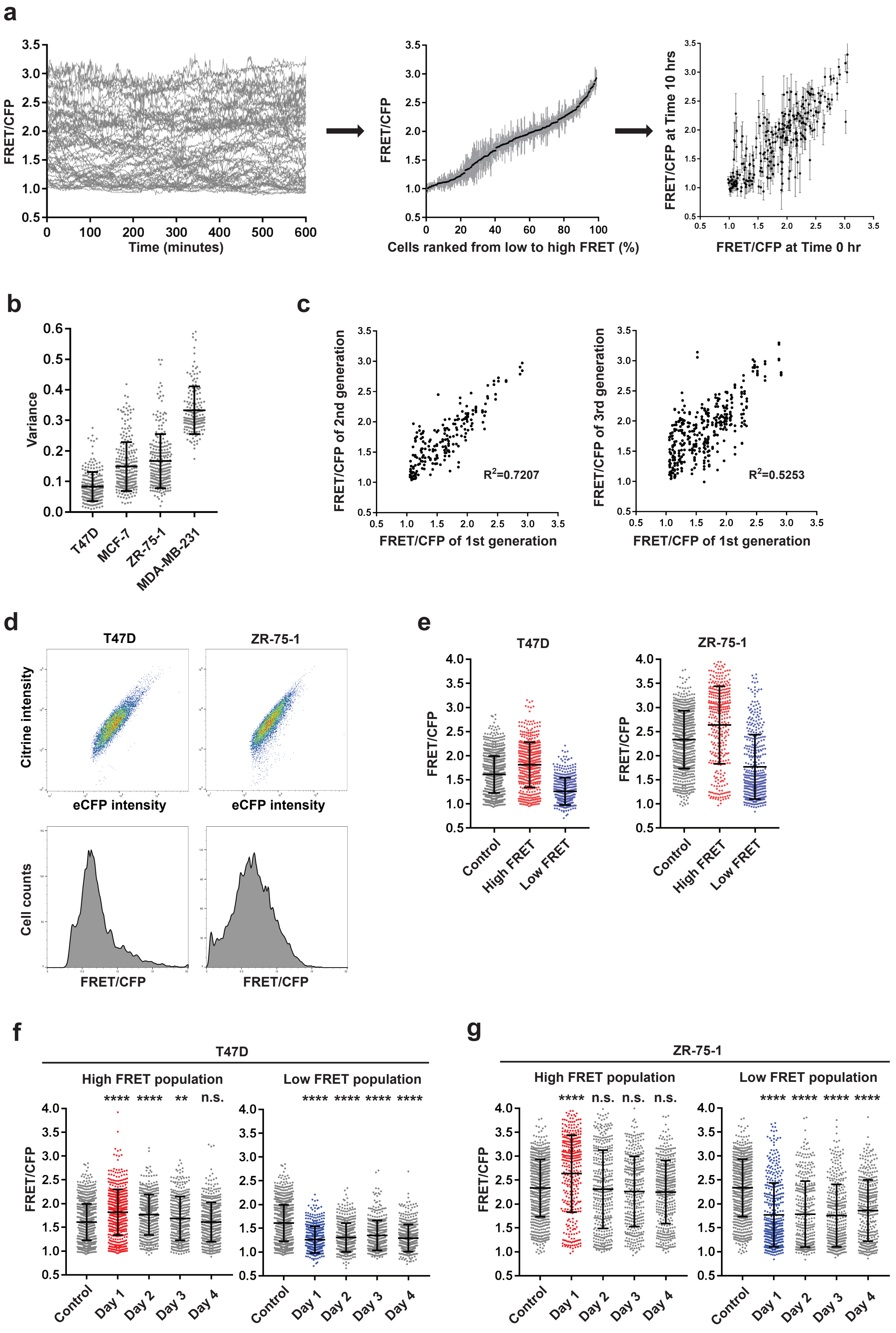
a
FRET/CFP
Time (minutes)
Cells ranked from low to high FRET (%)
FRET/CFP at Time 10 hrs
FRET/CFP at Time 0 hr
b
Variance
T47D
MCF-7
ZR-75-1
MDA-MB-231
c
FRET/CFP of 2nd generation
FRET/CFP of 1st generation
R2=0.7207
FRET/CFP of 3rd generation
FRET/CFP of 1st generation
R2=0.5253
d
T47D
ZR-75-1
Citrine intensity
eCFP intensity
eCFP intensity
Cell counts
FRET/CFP
FRET/CFP
e
T47D
ZR-75-1
FRET/CFP
Control
High FRET
Low FRET
f
T47D
High FRET population
Low FRET population
**** **** ** n.s.
**** **** **** ****
FRET/CFP
Control
Day 1
Day 2
Day 3
Day 4
g
ZR-75-1
High FRET population
Low FRET population
**** n.s. n.s. n.s.
**** **** **** ****
FRET/CFP
Control
Day 1
Day 2
Day 3
Day 4

Figure S2. Heritability and transitions between high and low glucose states. Figure S2 is related to Figure 2.

(A) Analysos of intracellular glucose level stability. Glucose biosensor-expressing breast cancer cell lines were imaged every 30 seconds for 10 hours. Following cell segmentation, glucose levels of individual cells were measured from 10-hour time-lapse imaging. The cells were ranked from low to high FRET and then glucose FRET signal at T = 0 hour against signal at T = 10 hours plotted with the standard deviation over 10 hours. **(B)** Variance of FRET distribution in glucose FRET biosensor-expressing breast cancer cell lines. n > 150 cells from at least three independent experiments. **(C)** Glucose FRET signals at 1st generation were plotted against signals at 2nd and 3rd generation. n > 100 cells from three independent experiments. **(D)** FRET distribution of intracellular glucose levels measured by flow cytometry. Panels show the FRET distribution of the glucose biosensor in T47D and ZR-75-1 cells. **(E)** Intracellular glucose levels of FACS-isolated high and low glucose concentration cells. FRET images were acquired 24 hours after cell isolation. n > 350 cells from three independent experiments. **(F, G)** Stability and heritability of FACS-isolated high and low glucose T47D and ZR-75-1 cells. High and low glucose populations were isolated and intracellular glucose levels were traced for 4 continuous days. n > 300 cells from three independent experiments. Data are shown as mean ± SD. Statistical significance was examined by Kolmogorov-Smirnov test. P values are indicated by ns (p > 0.05), ** (p < 0.01), **** (p < 0.0001).

Figure S3

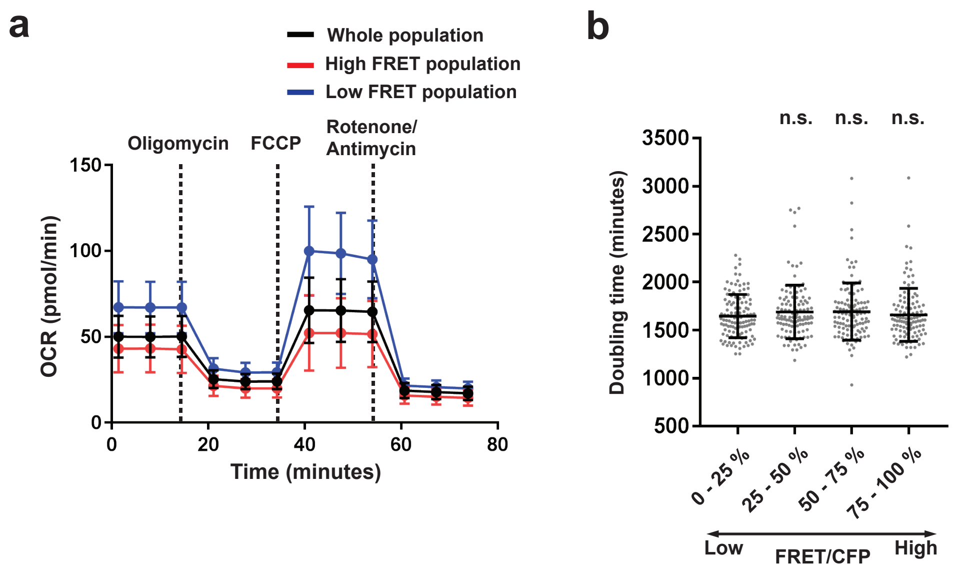

Figure S3. High and low intracellular glucose concentration indicates relative preference of glycolysis and OXPHOS. Figure S3 is related to Figure 3.

(A) OCR measurement of FACS-isolated high and low glucose concentration cells. OCR was measured every 6 minutes by adding Oligomycin, FCCP, and Rotenone/Actinomycin to a final concentration of 1, 0.5 and 0.5 µM respectively. n > 30 wells from three independent experiments. **(B)** Doubling time of MCF-7 cells in low to high glucose cells. MCF-7 cells were cultured in 0 mM Pyruvate DMEM and doubling time was measured in individual cells. Data are shown as mean ± SD. Statistical significance was examined by Kolmogorov-Smirnov test. P values are indicated by ns (p > 0.05).

Figure S4

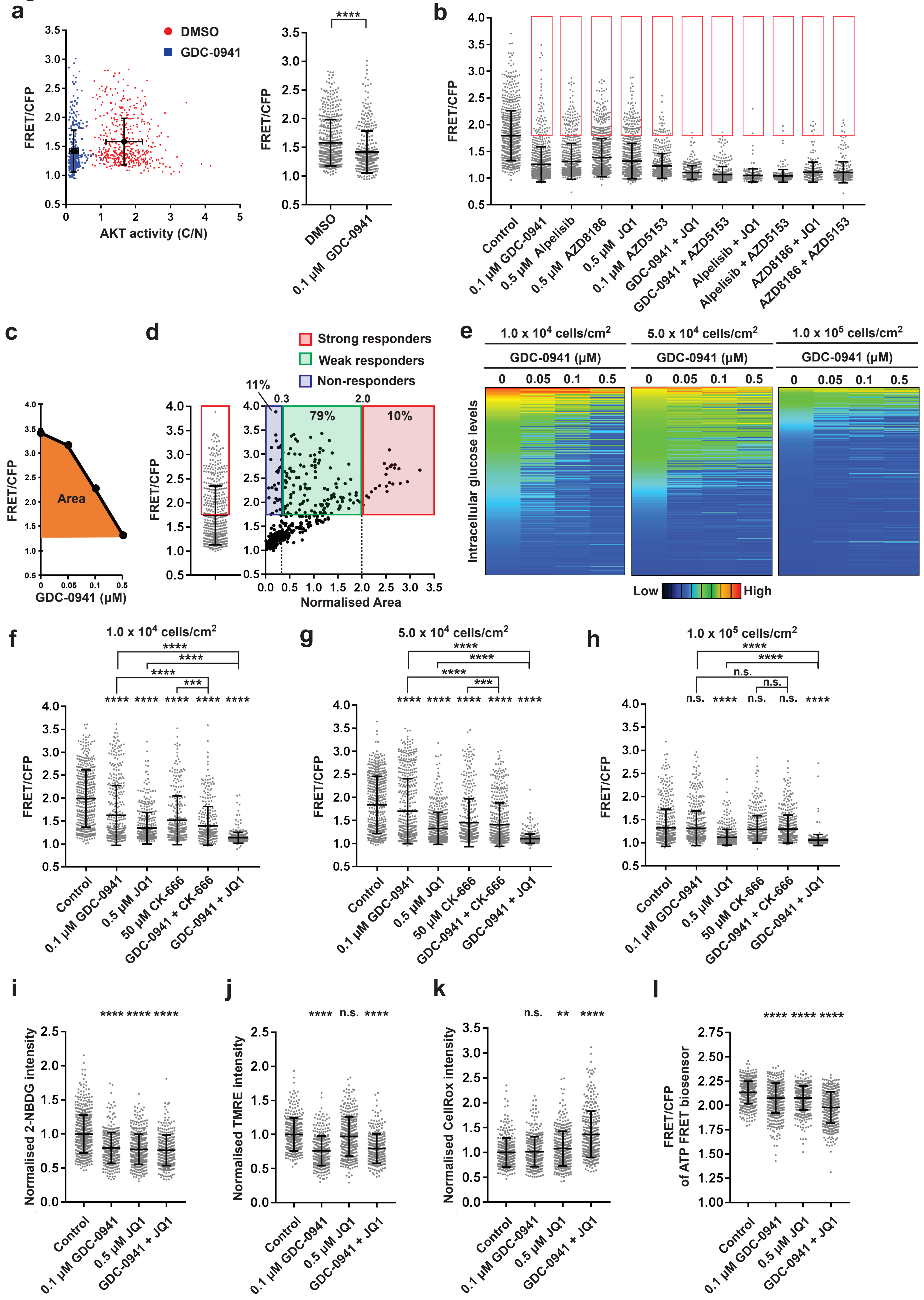

a
DMSO
GDC-0941
FRET/CFP
AKT activity (C/N)
****
0.1 µM GDC-0941
b
Control
0.1 µM GDC-0941
0.5 µM Alpelisib
0.5 µM AZD8186
0.5 µM JQ1
0.1 µM AZD5153
GDC-0941 + JQ1
GDC-0941 + AZD5153
Alpelisib + JQ1
Alpelisib + AZD5153
AZD8186 + JQ1
AZD8186 + AZD5153
c
Area
GDC-0941 (µM)
d
Strong responders
Weak responders
Non-responders
11%
79%
10%
Normalised Area
e
Intracellular glucose levels
Low
High
f
g
h
0.5 µM JQ1
50 µM CK-666
GDC-0941 + CK-666
i
Normalised 2-NBDG intensity
j
Normalised TMRE intensity
k
Normalised CellRox intensity
l
FRET/CFP of ATP FRET biosensor
n.s.

Figure S4. Inter-cellular heterogeneity in response to PI3K inhibition depends upon bromodomain function. Figure S4 is related to Figure 4.

(A) Multiplexed imaging of AKT and glucose FRET biosensor in MCF-7 cells. MCF-7 cells were incubated with 0.1 µM GDC-0941 for one hour. FRET ratio of the glucose FRET biosensor was plotted against the Cytoplasm/Nucleus (C/N) ratio of AKT biosensor. **(B)** FRET distribution of intracellular glucose levels in MCF-7 cells after PI3K and/or Bromodomain inhibitor treatments. MCF-7 cells were treated with 0.1 µM GDC-0941, 0.5 µM Alpelisib, 0.5 µM AZD8186, 0.5 µM JQ1, and 0.1 µM AZD5153. The cells indicated by the red square represent inhibitor resistant high glucose concentration cells. n > 200 cells from three independent experiments. Data presented for control, GDC-0941, JQ1 and GDC-0941 + JQ1 are also presented in Figure 4J and are included here for ease of comparison **(C)** Schematic representation of the calculation of area under the curve of cells responding to different concentrations of GDC-0941. **(D)** Non- (<0.3 in normalised area), weak- (0.3-2.0 in normalised area), or strong- (>2.0 in normalised area) responders to GDC-0941 are defined from normalised area under the curve responding to PI3K inhibitor. **(E)** Heat map of the FRET ratio of MCF-7 cells in different cell confluence and GDC-0941 concentrations. **(F-H)** The effect of combinatorial inhibition of PI3K, Arp2/3 complex, and Bromodomain on intracellular glucose levels of MCF-7 cells in different cell confluence. MCF-7 cells were treated with 0.1 µM GDC-0941, 50 µM CK-666, and 0.5 µM JQ1. n > 400 cells from three independent experiments. **(I-L)** The effect of combinatorial inhibition on energetic stress. Distribution of 2-NBDG intensity, TMRE intensity, CellRox intensity, and ATP FRET ratio in inhibitor treated MCF-7 cells. MCF-7 cells were incubated with 0.1 µM GDC-0941 and 0.5 µM JQ1 for 24 hours and then energetic conditions were analysed by imaging metabolic indicators. n > 250 cells from three independent experiments. Data are shown as mean ± SD. Statistical significance of 2-NBDG, TMRE, CellRox, and ATP FRET biosensor were examined by Kolmogorov-Smirnov test. P values are indicated by ns (p > 0.05), ** (p < 0.01), *** (p < 0.001), **** (p < 0.0001).

Figure S5

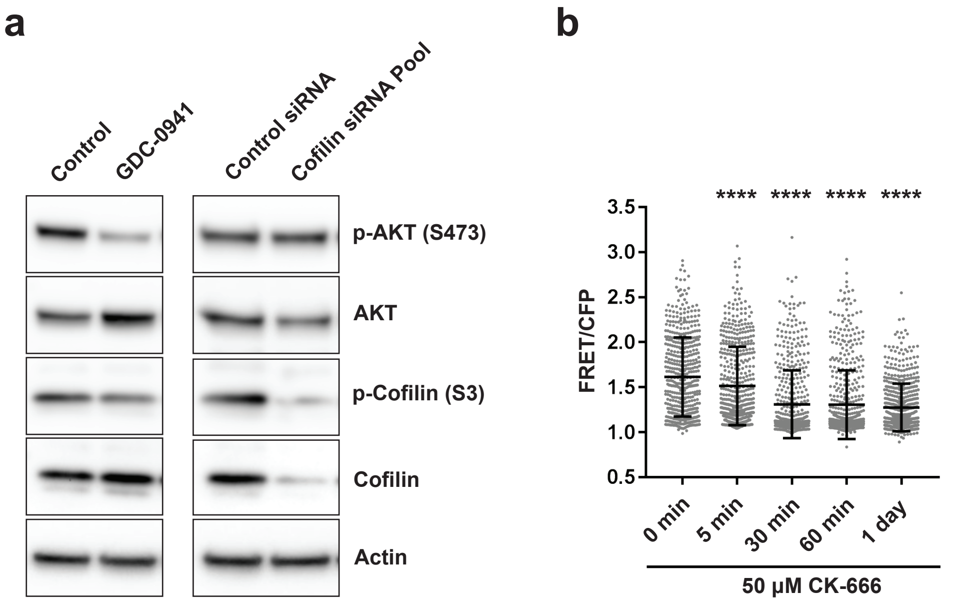

Figure S5. Confluence-dependent control of cofilin activity is a parallel regulator of intracellular glucose concentration. Figure S5 is related to Figure 5.

(A) Western blot of pAKT and pCofilin in PI3K inhibitor-treated and cofilin knock down MCF-7 cells.

(B) Time course imaging of intracellular glucose levels after Arp2/3 complex inhibitor treatment. n > 500 cells from three independent experiments. Data are shown as mean ± SD. Statistical significance was examined by Kolmogorov-Smirnov test. P values are indicated by **** ($p < 0.0001$).

Figure S6

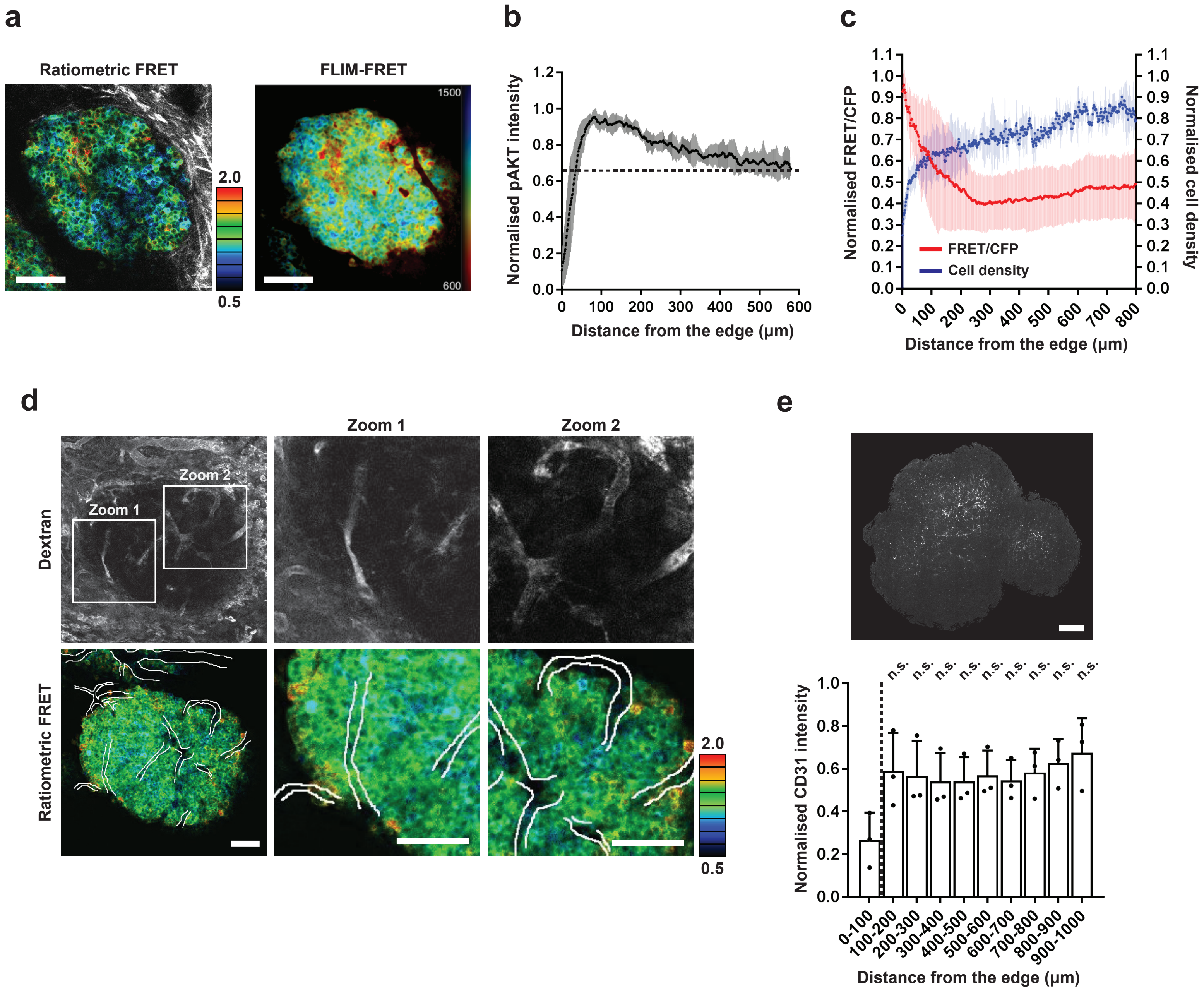

Figure S6. Intravital imaging reveals regional variation in metabolic state. Figure S6 is related to Figure 6.

(A) Intravital imaging of intracellular glucose levels of representative MCF-7 tumour in NOD SCID mouse. Intracellular glucose levels were measured by ratiometric FRET and FLIM-FRET. Scale of ratiometric FRET and FLIM-FRET = 100 µm. **(B)** Normalised phosphorylated AKT levels in MCF-7 tumours in the central-peripheral axis. **(C)** Normalised FRET ratio of glucose FRET biosensor and cell density of MCF-7 tumours in the central-peripheral axis. **(D)** Intravital imaging of intracellular glucose level and dextran of MCF-7 tumour in NOD SCID mouse. Dextran was injected to visualise blood vessels. Scale = 100 µm. Scale of high magnification image = 100 µm. **(E)** CD31 (PECAM-1) of resected tumours were immune-stained and quantified in the central-peripheral axis every 100 µm. Scale = 500 µm. Data are shown as mean ± SD. Statistical significance was examined by two-tailed unpaired t-test. P values are indicated by ns ($p > 0.05$).

Figure S7

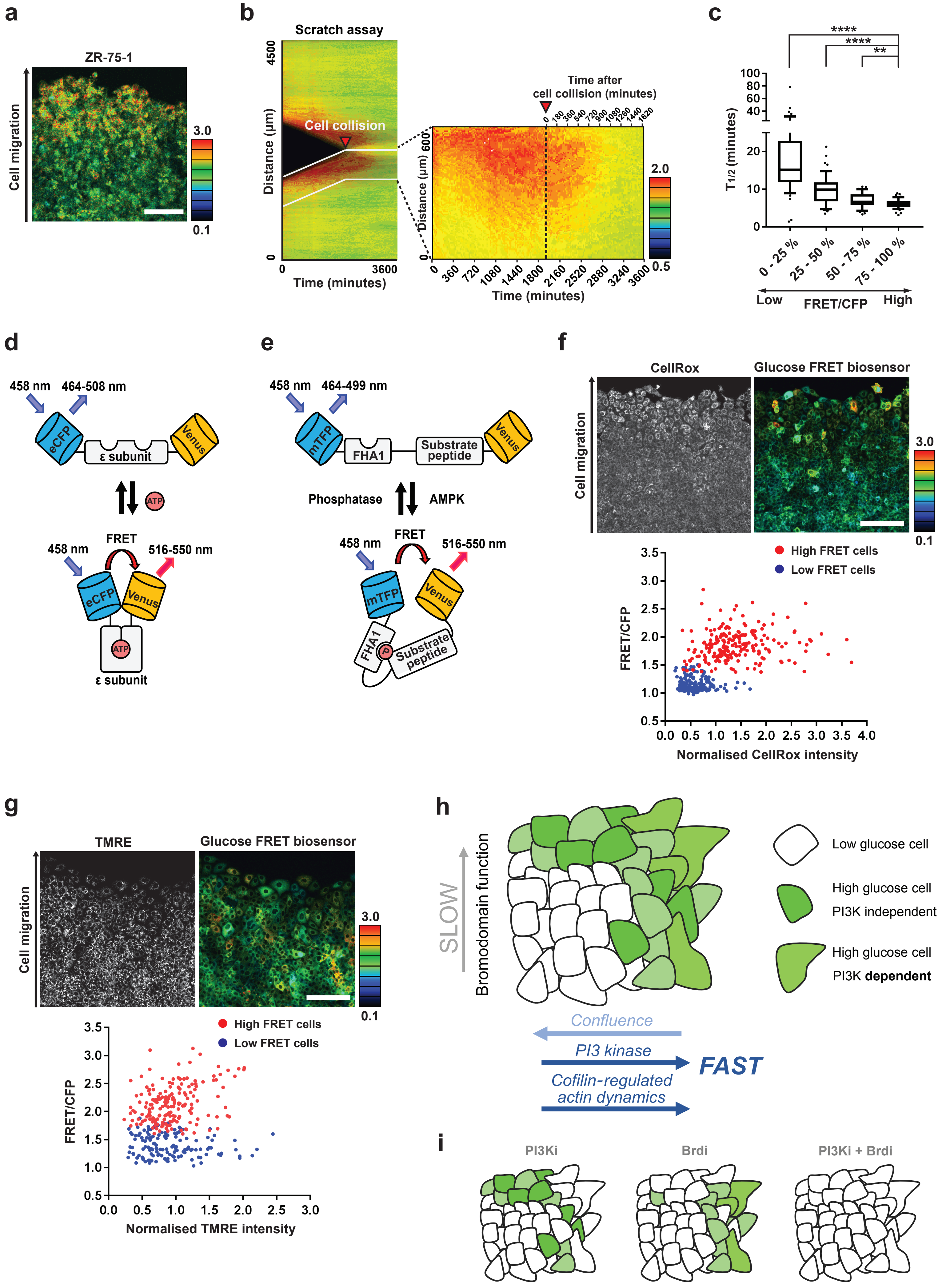

a
ZR-75-1
Cell migration
3.0
0.1
b
Scratch assay
Cell collision
Distance (µm)
4500
0
3600
Time (minutes)
Time after
cell collision (minutes)
0 180 360 540 720 900 1080 1260 1440 1620
Distance (µm) 600
0 360 720 1080 1440 1800 2160 2520 2880 3240 3600
2.0
0.5
c
****
****
**
T1/2 (minutes)
100
80
60
40
20
10
0
0 - 25 %
25 - 50 %
50 - 75 %
75 - 100 %
Low
FRET/CFP
High
d
458 nm
464-508 nm
eCFP
ε subunit
Venus
ATP
FRET
516-550 nm
ε subunit
e
458 nm
464-499 nm
mTFP
FHA1
Substrate peptide
Venus
Phosphatase
AMPK
FRET
516-550 nm
P
f
CellRox
Glucose FRET biosensor
Cell migration
3.0
0.1
High FRET cells
Low FRET cells
FRET/CFP
Normalised CellRox intensity
g
TMRE
Glucose FRET biosensor
Cell migration
3.0
0.1
High FRET cells
Low FRET cells
FRET/CFP
Normalised TMRE intensity
h
SLOW
Bromodomain function
Low glucose cell
High glucose cell
PI3K independent
High glucose cell
PI3K dependent
Confluence
PI3 kinase
FAST
Cofilin-regulated
actin dynamics
i
PI3Ki
Brdi
PI3Ki + Brdi

Figure S7. 'Scratch' assays recapitulate the regional variation in metabolic state. Figure S7 is related to Figure 7.

(A) Scratch assay of glucose FRET biosensor-expressing ZR-75-1 cells. Scale = 100 µm. **(B)** Kymograph of FRET signals in scratch assay of MCF-7 cells. Red arrow indicates the time point of cell collision. **(C)** Half-life of intracellular glucose levels to drop the baseline in scratch assay. Individual curves were fitted by one phase decay. Data are shown as box plots of median with lower to higher quartiles, and 10 to 90 percentile whiskers. **(D)** Schematic representation of the conformational shift of the AMPK FRET biosensor. The substrate peptide of the AMPK FRET biosensor is phosphorylated by AMPK and changes its conformational structure. **(E)** Schematic representation of the conformational shift of the ATP FRET biosensor. The ATP FRET biosensor binds with intracellular ATP and changes its conformational structure. **(F)** CellRox intensity in scratched glucose FRET biosensor-expressing MCF-7 cells. The cells were incubated with 5 µM CellRox 24 hours after the scratch. The FRET ratio of the glucose FRET biosensor and CellRox intensity were measured in high glucose concentration cells at the leading edge and low glucose concentration cells at an area of high cell density. CellRox intensity was normalised by cell area. n > 150 cells from three independent experiments. Scale = 200 µm. **(G)** TMRE intensity in scratched glucose FRET biosensor-expressing MCF-7 cells. The cells were incubated with 100 nM TMRE 24 hours after the scratch. The FRET ratio of the glucose FRET biosensor and TMRE intensity were measured in high glucose concentration cells at the leading edge and low glucose concentration cells at an area of high cell density. TMRE intensity was normalised by cell area. n > 100 cells from three independent experiments. Scale = 200 µm. **(H, I)** Schematic representation of intra-tumour heterogeneity in glycolysis, transitions between metabolic states, their heritability and regulatory mechanisms.

Data S1. Original western blot images. Related to Figures 4, 5, and S5.

Blots related to Figure 4a and 4j:

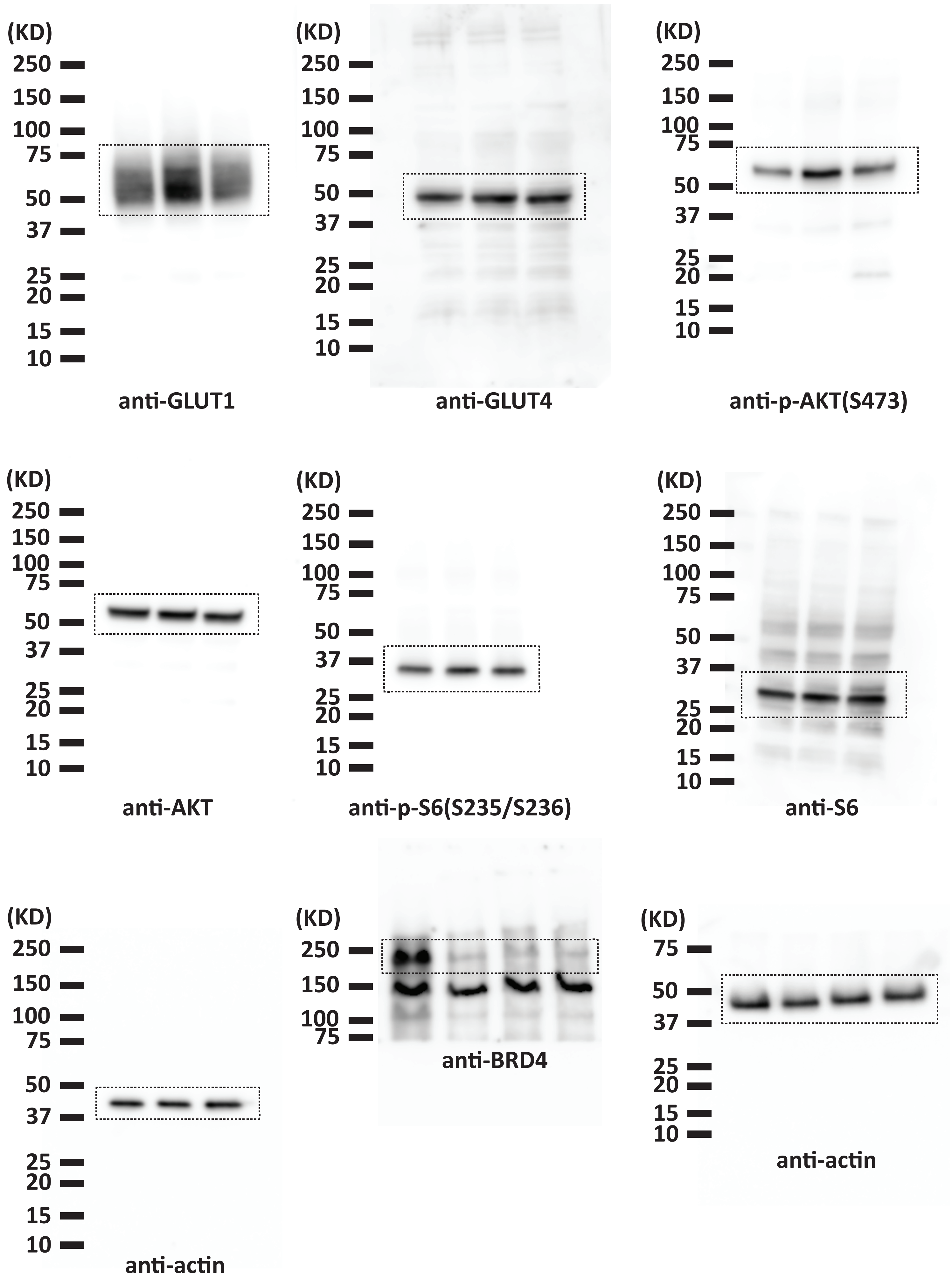

Blots related to Figure 5a, 5b, and 5d:

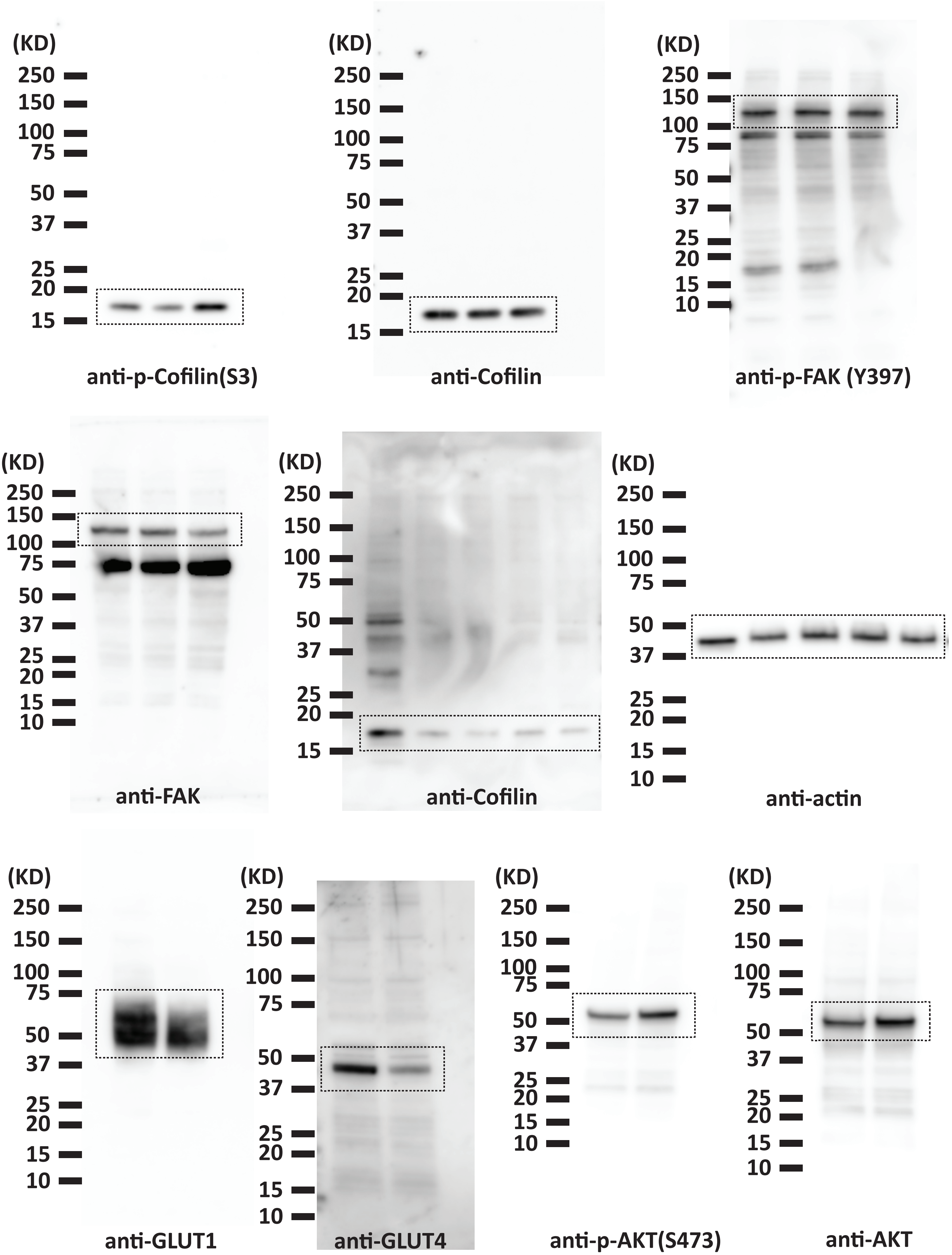

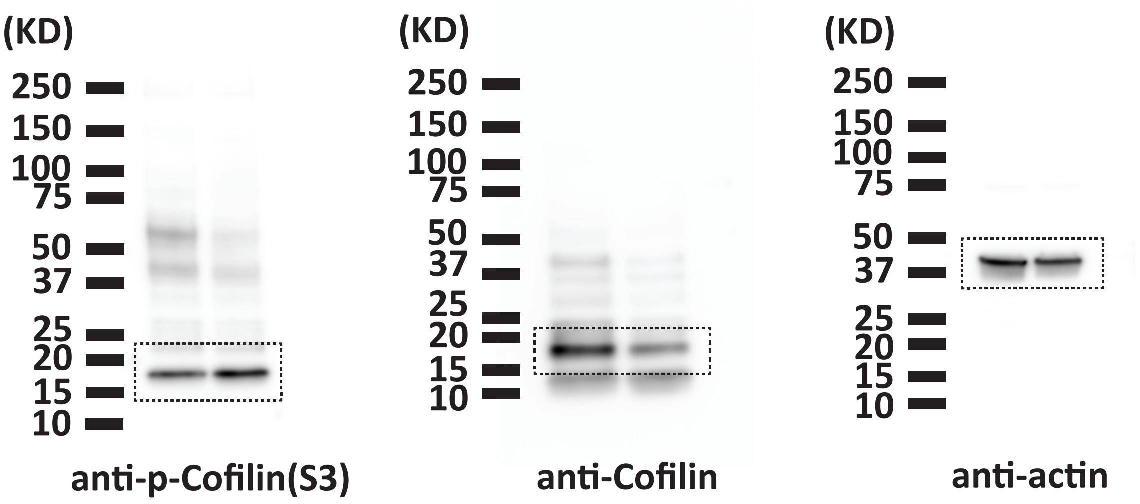
(KD)
250
150
100
75
50
37
25
20
15
10
anti-p-Cofilin(S3)
(KD)
250
150
100
75
50
37
25
20
15
10
anti-Cofilin
(KD)
250
150
100
75
50
37
25
20
15
10
anti-actin

Blots related to Figure S5a:

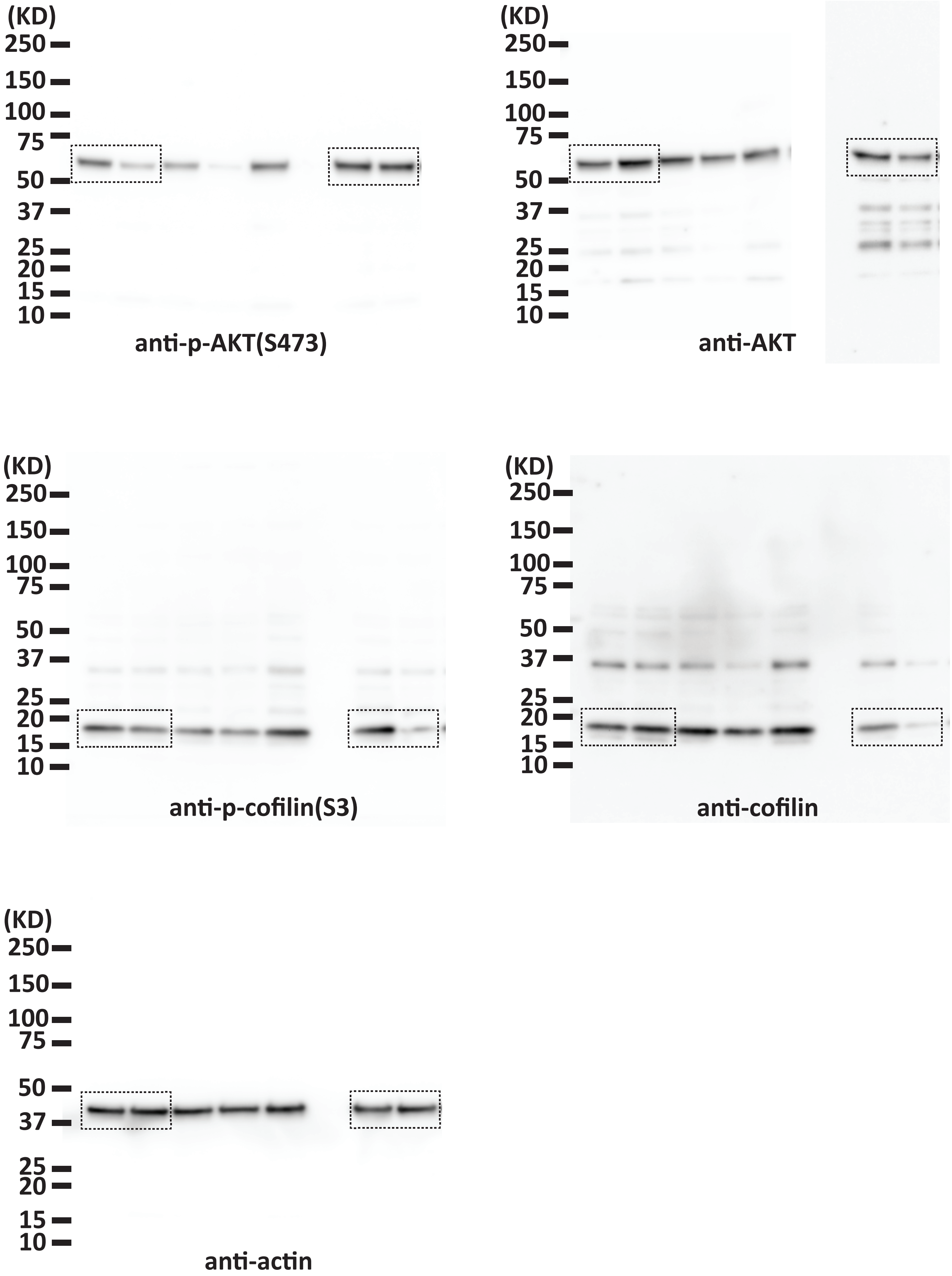

Supplement: Document S1. Figures S1–S7 [file mmc1.pdf]
